# Convergent evolution and topologically disruptive polymorphisms among multidrug-resistant tuberculosis in Peru

**DOI:** 10.1371/journal.pone.0189838

**Published:** 2017-12-27

**Authors:** Louis Grandjean, Robert H. Gilman, Tomatada Iwamoto, Claudio U. Köser, Jorge Coronel, Mirko Zimic, M. Estee Török, Diepreye Ayabina, Michelle Kendall, Christophe Fraser, Simon Harris, Julian Parkhill, Sharon J. Peacock, David A. J. Moore, Caroline Colijn

**Affiliations:** 1 University College London, Institute of Child Health, London, United Kingdom; 2 Academic Health Sciences Centre, Imperial College, London, United Kingdom; 3 Universidad Peruana Cayetano Heredia, Avenida Honorio Delgado, San Martin de Porras, Lima, Peru; 4 Wellcome Trust Sanger Institute, Genome Campus, Hinxton, Cambridge, United Kingdom; 5 Johns Hopkins Bloomberg School of Public Health, Baltimore, MD, United States of America; 6 Department of Infectious Diseases, Kobe Institute of Health, Chuo-ku, Kobe, Japan; 7 Department of Medicine, University of Cambridge, Cambridge, United Kingdom; 8 Faculty of Natural Sciences, Department of Mathematics, Imperial College London, London, United Kingdom; 9 Department of Infectious Diseases Epidemiology, Imperial College, London, United Kingdom; 10 London School of Tropical Medicine and Hygiene, London, United Kingdom; St Petersburg Pasteur Institute, RUSSIAN FEDERATION

## Abstract

**Background:**

Multidrug-resistant tuberculosis poses a major threat to the success of tuberculosis control programs worldwide. Understanding how drug-resistant tuberculosis evolves can inform the development of new therapeutic and preventive strategies.

**Methods:**

Here, we use novel genome-wide analysis techniques to identify polymorphisms that are associated with drug resistance, adaptive evolution and the structure of the phylogenetic tree. A total of 471 samples from different patients collected between 2009 and 2013 in the Lima suburbs of Callao and Lima South were sequenced on the Illumina MiSeq platform with 150bp paired-end reads. After alignment to the reference H37Rv genome, variants were called using standardized methodology. Genome-wide analysis was undertaken using custom written scripts implemented in R software.

**Results:**

High quality homoplastic single nucleotide polymorphisms were observed in genes known to confer drug resistance as well as genes in the Mycobacterium tuberculosis ESX secreted protein pathway, pks12, and close to toxin/anti-toxin pairs. Correlation of homoplastic variant sites identified that many were significantly correlated, suggestive of epistasis. Variation in genes coding for ESX secreted proteins also significantly disrupted phylogenetic structure. Mutations in ESX genes in key antigenic epitope positions were also found to disrupt tree topology.

**Conclusion:**

Variation in these genes have a biologically plausible effect on immunogenicity and virulence. This makes functional characterization warranted to determine the effects of these polymorphisms on bacterial fitness and transmission.

## Introduction

The World Health Organization estimates that multidrug-resistant tuberculosis causes 500 deaths and 1300 new infections each day [1]. Understanding the genetic basis of tuberculosis drug resistance, host immune evasion and bacterial phenotype is important to inform the development of new diagnostic, treatment and preventive strategies. Identifying convergent evolution in multidrug-resistant tuberculosis may uncover how *Mycobacterium tuberculosis* is adaptively evolving to evade host immunity and antibiotic chemotherapy. Determining which variant sites are most disruptive of phylogenetic structure could also uncover important genotypic influences on phenotype.

Homoplasy is defined as the emergence of identical traits or characters occurring independently in different clades that are not present in their common ancestor [2]. Homoplastic events are often associated with adaptive advantages, a frequently cited example being the independent evolution of the eye across multiple different species [3]. Analyses of genome wide data looking for homoplasious signals have already led to the identification of genes associated with echo location in mammals [4], caffeine production in coffee and tea [5], and the adaptation of *Pseudomonas* to the human lung in cystic fibrosis [6]. Homoplastic mutations among drug resistant *M*. *tuberculosis* may code for drug resistance or mechanisms of immune subversion [7,8].

Only a few studies have examined *M*. *tuberculosis* strain collections for evidence of homoplasy. Casali et al [9] identified a set of homoplastic mutations among a large collection of 1000 prospectively collected strains in Samara Oblast, while Farhat et al [7] in a smaller dataset of 123 strains compared the occurrence of multiple independent mutations in MDRTB strains to that among drug susceptible strains. Others have screened a selected set of genes encoding surface proteins for homoplasy to test the hypothesis that mutations in these surface proteins at the interface with the human immune system lead to significant adaptive advantage [8]. No studies have examined the correlation between homoplastic sites or identified variant sites that particularly influence phylogenetic structure.

In order to identify homoplasy, topologically disruptive polymorphisms and evidence of epistasis we sequenced the genomes of 471 predominantly multidrug-resistant tuberculosis isolates collected in the suburbs of metropolitan Lima, Peru.

## Results and discussion

**Fig 1:**
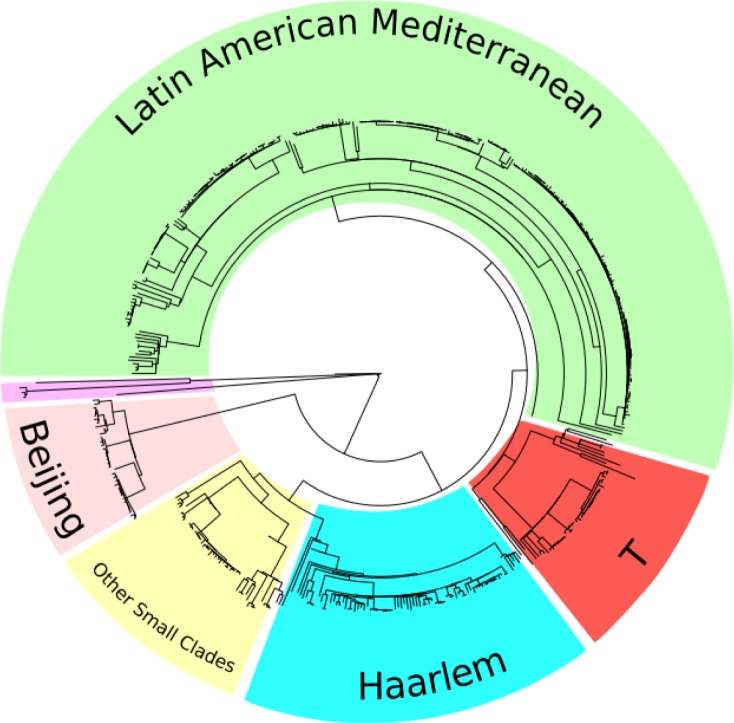
The whole genome maximum likelihood SNP based phylogeny of 471 study strains together with the reference H37Rv and *Mycobacterium canettii*.

Patient demographics. The median age of recruited patients was 30 years (IQR 23–42) with an HIV prevalence of 4% (19/469) and a smear positivity percentage of 90% (416/469), S1 Table. The 471 sequences clearly clustered phylogenetically into 5 main groups together with the out-group of Mycobacterium canetti and 3 circulating strains of M. caprae observed at the population level in Lima (Fig 1) supported by high (>99%) bootstrap probabilities and also by principle component analysis. Samples were predominantly from lineage 4 (Euro-American) which comprised 54% (255/469) LAM (Latin American Mediterranean), 17% (81/469) Haarlem, 10% (47/469) T Clade and 10% (47/469) other small clades (including X, S and other T-type MIRU-spoligotypes). A total of 34 (7%) were from the lineage 2 Beijing family and the remainder of the dataset included 3 Mycobacterium caprae strains, one Mycobacterium bovis strain and one East Asian Indian Manilla (EIA2) strain (Table 1).

**Table 1:** Table of demographics.

	Number	Proportion
Whole Genomes Sequenced	471	100%
Metadata Available (Denominator)	469	99%
Sex		
Male	288	61%
Female	179	38%
Unknown	2	1%
Age Median (IQR) Overall	0 (23–42)	-
<10	0	0%
10–<20	49	10%
20–<30	174	37%
30–<40	104	22%
40–<50	54	12%
50–<60	38	8%
>60	37	8%
Unknown	13	3%
Ziehl Neelsen Smear Status		
Positive	416	89%
Negative	46	10%
Unknown	7	1%
Previous TB Disease		
Yes	298	64%
No	171	36%
Unknown	0	0%
HIV Status		
Positive	19	4%
Negative	450	96%
Unknown	0	0%
Drug Resistance Profile		
Susceptible^1^	26	6%
Rifampicin Resistant	33	7%
Isoniazid Resistant	97	21%
Multidrug Resistant	311	66%
MIRU-Spoligotype Available	240	52%
Clade		
Latin American Mediterranean	255	54%
Haarlem	81	17%
Beijing	34	7%
T	47	10%
*Mycobacterium caprae*	3	<1%
*Mycobacterium bovis*	1	< 1%
East Asian Indian	1	<1%
Other Small Clades^2^	47	10%

^1^Susceptible to Rifampicin and Isoniazid.

^2^Comprised the MIRU defined ‘S’ family, ‘X’ family and ‘T’ family strains.

### Phylogenetic comparison of 15-loci MIRU-VNTR with whole genome sequencing

**Fig 2:**
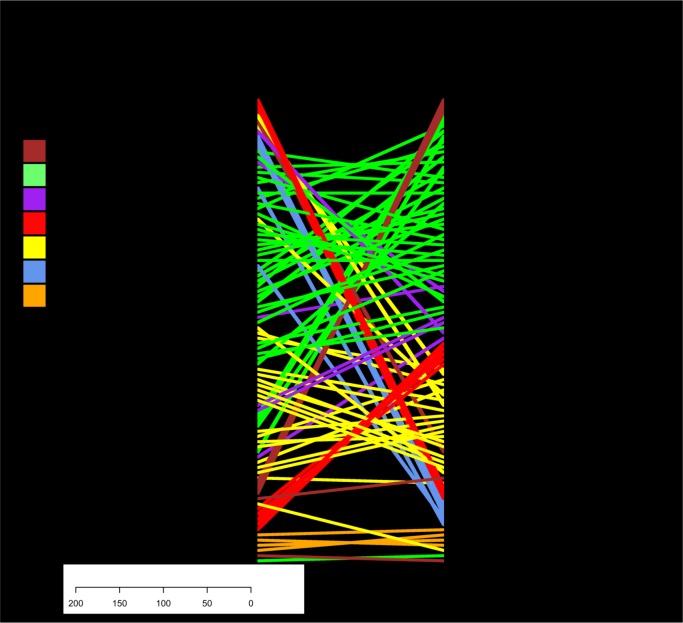
A 15-loci MIRU-VNTR derived neighbour joining tree (right) of 142 strains of the LAM clade (lineage 4) for which both whole genome sequence data were available labelled according to Institut Pasteur website www.miru-vntrplus.org and compared to a whole genome sequence neighbour joining tree (left) demonstrating the misclassification of strains into groups at high definition based on MIRU alone.

At high resolution when the sub-clades of the LAM clade as defined by MIRU-VNTR were compared to whole genome sequence defined clades (Fig 2), significant disagreement in clade topology was observed. This highlights the unreliable nature of defining a sub-clade based on MIRU-VNTR alone. MIRU derived trees correlated better with the whole genome trees than trees constructed from concatenated MIRU-spoligotypes and much better than trees made from spoligotyping alone (S1 Fig).

### Homoplastic non-synonymous polymorphisms

**Fig 3:**
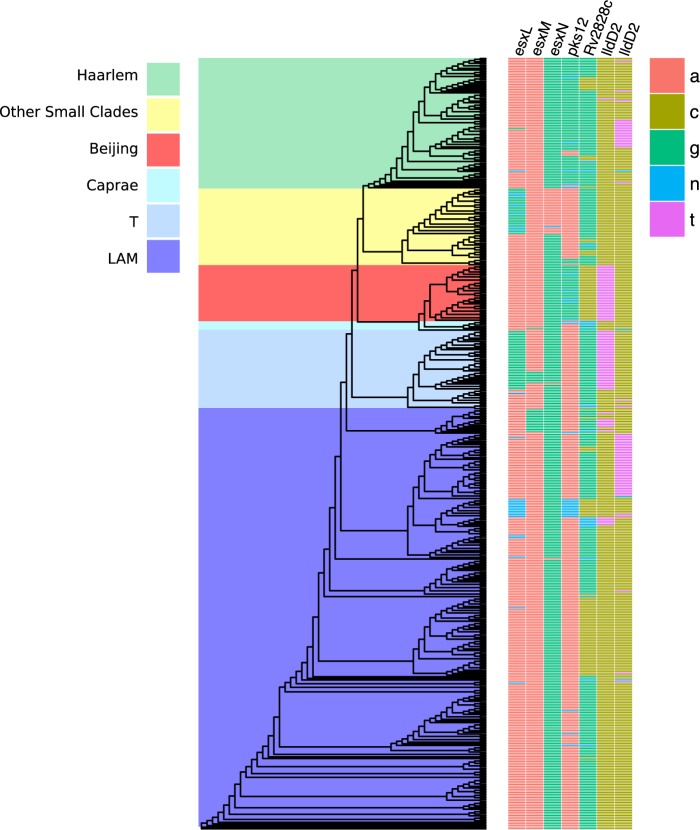
Phylogeny of study strains with clade names and position of homoplastic sites identified in the study.

Many of the known drug resistance mutations were observed to occur homoplastically across the phylogeny (S2 Table). These mutations have been widely reported elsewhere [10]. A phylogeny of the study strains together with the sites of homoplastic polymorphisms is provided in Fig 3.

The *Rv2828c* non-synonymous mutation Thr141Met is particularly interesting as a separate intergenic homoplastic polymorphism was also identified in the promoter region 2bp upstream of the start of *Rv2828c* (position 3136343). This gene has not as yet been implicated with drug resistance or virulence, however the *Rv2828c* gene is adjacent to a toxin antitoxin (TA) system *vapC22* (*Rv2829c*) *vapB22* (*Rv2830c*) which has been demonstrated to limit growth of *M*. *smegmatis* [11]. Toxin gene products of *vapB* and *vapC* block *M*. *tuberculosis* translation via RNA cleavage thereby slowing down the replication rate facilitating successful latent infection [11,12]. Many antibiotics target bacterial growth, making slowly replicating bacteria more refractory to treatment [13]. Ramage et al highlight that the significant expansion of TA systems relative to the last common ancestor of *M*. *tuberculosis* suggesting an important role for these systems in *M*. *tuberculosis* evolution [11].

The non-synonymous homoplastic polymorphism in *esxI* occurred in the epitope coding region at position 1160767 (Ser23Leu). This polymorphism has been described by Upleker at al [14] as critical to immunogenicity and therefore likely to confer a functionally beneficial adaptive advantage.

The *lldD2* non-synonymous single nucleotide polymorphism Val3Ile (at position 2123153) occurred independently 12 times and expanded to a total of 70 strains at the tips of the tree (a second non-synonymous homoplastic *lldD2* Val253Met variant at position 2122403 was also identified). The *lldD2* gene is a putative lactate dehydrogenase. Osorio et al [8] identified this polymorphism when evaluating genes encoding membrane proteins for diversifying selection. They speculated that mutations in this gene could represent a metabolic adaptation to host environment such as anaerobic conditions. One synonymous polymorphism also occurred homoplastically in *Rv1873*, a gene of unknown function located adjacent to *lldD2* [15].

### Homoplastic intergenic polymorphisms

The most homoplastic intergenic polymorphism (occurring 13 times independently and expanding to 64 strains at the tips of the tree) arose 15bp upstream (position 1673433) of *fabG1* in the promoter region [16]. This gene is associated with isoniazid resistance as it can act as an alternative promoter for *inhA* [17].

### Homoplastic synonymous polymorphisms in ESX genes

Emerging evidence suggests that synonymous sites may also be under selection because of adaptive changes in gene expression via transcription factor binding or mRNA stability [18]. The ESAT-6 like ESX proteins were over represented among the synonymous polymorphisms identified in this analysis. The family of proteins coded by these genes are the most immunodominant of *M*. *tuberculosis* antigens. A synonymous *esxK* mutation (position 1340675 A to G) occurred independently on 9 occasions expanding to 84 strains at the tips of the tree. This mutation fell in the middle of a transcription factor binding hotspot that has been demonstrated to bind 10 different gene regulators [19]. Only 2.5% of the genome acts as a binding site for multiple transcription factors making these regions highly likely to influence gene expression. Supporting the importance of the esx genes in immunogenicity, Villareal and colleagues [20] have already demonstrated substantial antigen-specific IFN-γ spot-forming cells to all esx antigens together with a maintained memory response. In addition, none of *esxO*, *esxV*, *esxP*, *esxW*, *esxA*, and *esxB* are present in avirulent BCG [21]. The conservation of silent as well as nonsynonymous SNPs between paralogs and orthologs of the ESX family, as seen in *esxP* and *esxM*, respectively, suggests that even minor variation within these families could significantly alter the expression of these proteins [14]. Uplekar et al also suggested that sequence changes in ESX genes are likely to lead to immune variation and found evidence of intra-genomic recombination as a potential source of variation in these genes. Skjot and colleagues hypothesized that the amino acid substitutions encoded by the duplicated genes for the ESAT-6 protein family may allow for antigenic drift, wherein the regulated expression of functionally similar protein homologs that differ in their immunodominant epitopes result in antigen variation and immune system escape [22].

Other homoplastic synonymous polymorphisms that occurred in transcription factor binding hotspots included those in *esxL* (3 homoplastic sites, C>G at position 1341052), *esxO* (2 homoplastic sites, C>G at position 2626103) and Rv1873 (a gene upstream of lldD2, 3 homoplastic sites, A>C at position 2123190). The genes *esxL*, *esxM* and *esxN* also demonstrated synonymous polymorphism homoplasy albeit with two ancestral homoplastic mutations only. Two homoplastic synonymous sites were identified in the gene *pks12* also identified as homoplastic by Farhat et al [7]. This gene is the largest in the *Mycobacterium tuberculosis* genome and is involved in pathogenesis by dimycocerosyl phthiocerol synthesis [23].

The esx genes also featured in the phyC output (S3 Table) with mutations in *esxK*, *esxN* and *esxV* also identified as being homoplastic. The homoplastic synonymous polymorphism in *esxN* identified by phyC also occurred in the epitope coding region of *esxN* at position 2030950.

Homoplastic mutations in *lldD2*, *pks12*, *Rv2828c*, *Rv0277* were also confirmed with this technique. Additional homoplastic mutations of interest identified with phyC but not with the accelerated transformation analysis included the mutation occurring in *Rv2571c*, a gene located adjacent to *aspS*, which is involved in the *M*. *tuberculosis* translational pathway.

### Correlation between homoplastic sites

Blocks of correlation indicative of epistatic interaction were identified between homoplastic polymorphisms (S2 Fig). Notably the homoplastic mutations in *rpsL*, *Rv2082*, *lppB*, *lldD2* and the intergenic region around position 3841670 were all significantly positively correlated. Similarly, the *pks12* gene mutation was positively correlated with mutations in *Rv2082* and intergenic regions 3067969, 3122583, 3232711 and 3820553. A strong negative correlation was observed between mutations in *pks12* and *Rv2082* as well as between *esxV* with intergenic mutation at position 3738660. Unexpectedly no correlation was observed between *rpoC* mutations and rpoB mutations. Mutations in *rpoC* have been demonstrated to compensate for mutations in *rpoB* in vitro and therefore we hypothesized that homoplastic mutations in these two genes may be correlated [24]. A similar lack of correlation was seen in other well documented drug resistance mutations (*katG*, *gyrA*, *inhA*) suggesting that there is not a homoplastic compensatory mechanism for the drug resistance mutations in these genes.

**Fig 4:**
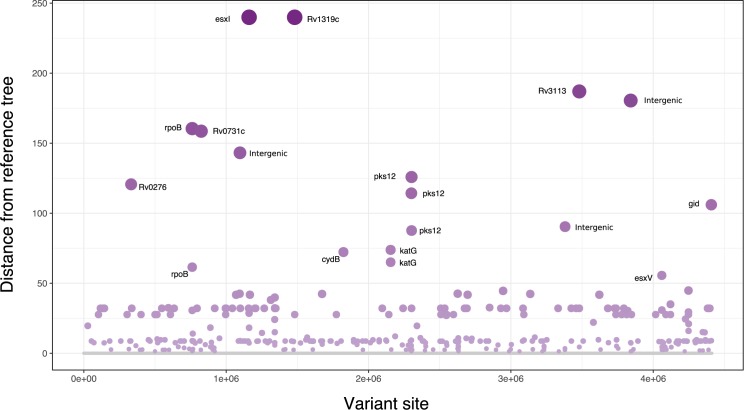
Manhattan plot of the polymorphisms most influential of phylogenetic structure with the most significant genes labelled.

Topologically disruptive sites. Only 2.19% (382/17476) of all variant sites were found to disrupt phylogenetic structure (S4 Table, Fig 4). The variant site that most affected phylogenetic structure was a non-synonymous single nucleotide polymorphism observed in *esxI* at position 1160776 (Gln20Leu). This polar to non-polar substitution occurred right in the middle of the *esxI* epitope coding region making it likely to influence the immune response to *M*. *tuberculosis* infection. The paralogous nature of the esxI gene with repeating regions up and down stream does however make this mutation less reliable when called by 150bp read sequencing. It is therefore necessary to repeat and confirm this observation using longer read sequencing platforms. The tree difference algorithm also identified the non-synonymous Leu23Ser mutation at the antigenically critical position in the middle of the epitope region of *esxL* at position 1341081. Once again this was not picked up by either Sanger or phyC homoplasy software. This supports our hypothesis that phylogenetically disruptive polymorphisms may affect phenotype; three of the fifty most informative sites also included *rpoB*, *katG* and *embB* mutations, well documented to have phenotypic consequence in drug resistance.

Genome wide association corrected for phylogenetic structure by principal component analysis

Correcting for principal components significantly reduced the background noise in the Manhattan plots demonstrating the key polymorphisms involved in second line drug resistance (S3 and S4 Figs). Genome wide analysis for sputum smear grade (positive vs negative), gender, HIV status and previous treatment did not uncover any significant polymorphisms.

### Conclusion

This study has identified the presence of a set of convergent homoplastic polymorphisms among a collection of 471 predominantly multidrug-resistance tuberculosis strains in Peru. Homoplasy is highly associated with beneficial adaptive evolution as evidenced by the confirmation of many homoplastic drug resistance mutations and mutations in the highly immunogenic secreted protein ESX gene family. Mutations in the ESX genes may therefore have implications for vaccine development, while designing drugs to target these gene products may help to prevent the consequences of adaptive evolution or immune evasion of multi-drug resistant *M*. *tuberculosis*. A non-synonymous Leu23Ser mutation at the antigenically critical position in the middle of the epitope region of *esxL* at position 1341081 was identified by the tree difference algorithm. This lends weight to our hypothesis that polymorphisms that affect tree topology can have phenotypically significant consequences. Functional and longer sequencing read confirmation of the homoplastic and topologically disruptive polymorphisms identified here is therefore warranted as well as the use of this technique to identify informative sites in other organisms.

## Materials and methods

Ethics approval and consent to participate. Ethical approval for sample collection and processing was obtained from the IRB of the Universidad Peruana Cayetano Heredia as part of previously published studies [25,26] and institutional approval was obtained from the Peruvian Ministry of Health. Individual patient consent was not sought as the data was collected and analysed anonymously.

### Field methods, culture techniques and sample selection

Collection of patient metadata, sputum samples, culture techniques, DNA extraction, MIRU typing and spoligotyping were undertaken as previously described [25,26]. Briefly, samples were selected from two large studies undertaken in the regions of Callao (population size 800,000) and Lima South (population size 1,200,000). The first study (“population level study”) undertaken between 2008–2010 sampled all patients presenting to tuberculosis clinics and hospitals in these areas as part of the population level implementation of Microscopic Observed Drug Susceptibility (MODS) testing [27,28]. The second study (“household follow-up study”) followed 213 households with an MDRTB index case and 487 households with a DSTB index case in the same study area over a period of 3 years between 2010–2013.

### Population level study sampling

Samples were collected from 2086 unselected unique patients presenting with symptoms of tuberculosis across this study area. All study samples were genotyped with 15-loci MIRU-VNTR and spoligotyping. At least one strain was selected for whole genome sequencing from every MIRU-VNTR and spoligotype defined cluster in order to sample a representative selection of total population genetic diversity, maximize genomic variability and to improve analytical power. A total of 198 samples were selected from MIRU-spoligotype defined clusters as well as 87 samples from unique MIRU-spoligotypes. Metadata was gathered using a structured questionnaire that was completed at the time of sputum collection.

### Household study sampling

The second study recruited unselected newly diagnosed multidrug-resistant tuberculosis patients in the same study areas as part of a 3-year long household follow up study conducted between 2010–2013. This study recruited a total of 213 MDRTB patients of which 186 multidrug-resistant tuberculosis strains were selected at random to contribute to this analysis. Metadata was collected in a structured questionnaire completed at recruitment for household follow-up.

All tuberculosis patients in Peru are tested for HIV, so this data was available from the patient records at the time of recruitment to both studies. Sputum samples from all patients in both studies were transported to the regional reference laboratories and processed both on liquid (MODS) and solid Ogawa media. An aliquot of each positive culture was sub-cultured at Universidad Peruana Cayetano Heredia and spoligotyping was performed [29] after DNA extraction [30]. DNA was sent to the Kobe Institute, Japan for 15-loci MIRU VNTR typing [31]. Any drug resistant sample (resistant to rifampicin or isoniazid) based on MODS was retested at the national reference laboratory using the proportions method on agar.

### Genome sequence quality control

We prepared Illumina sequencing libraries with a 450 bp insert size, using instructions in the manufacturer’s protocols, and then undertook sequencing on an Illumina HiSeq2000 with paired-end reads of length of 100 bp. To this end we multiplexed 96 samples per lane to attain an average depth of coverage of ~ 97.17-fold. We confirmed the species in the short reads using Kraken [32]. We assembled paired end sequence reads with an improved assembly pipeline [33], based on Velvet [34]. A list of isolates and their accession numbers in the European Nucleotide Archive is provided in S4 Table (project number: ERP004677). We mapped short reads to the corrected H37Rv reference genome available from Casali et al. [35] genome.cshlp.org/content/suppl/2012/02/01/gr.128678.111.DC1/1_H37RvQM_embl.txt. In doing so, we employed SMALT v0.7.4 (www.sanger.ac.uk/science/tools/smalt-0) using maximum and minimum inserts sizes of 1000 and 50, respectively. To annotate SNPs, we used SAMtools mpileup [36] and BCFtools, as it is described by Harris et al [37]. We included SNPs that were covered by at least two forward and two reverse short paired end reads [38]. A minimum base call quality of 50 and a minimum root mean squared mapping quality of 30 to call a SNP were used. Furthermore, the SNPs at sites with heterogeneous mapping where less than 75% of reads at that site covered the SNP were excluded from the analysis [37]. We obtained the multiple alignment by generating pseudo-sequences, after ignoring the small indels.

### Phylogenetic analysis and ancestral state reconstruction

Maximum likelihood, parsimony and neighbour joining phylogenies were constructed with concatenated SNPs from the whole genome sequence data using R software (R Foundation for Statistical Computing, Vienna, Austria 2011, www.R-project.org) with packages “adegenet”, “phangorn” [39] as well as RAxML [40]. Whole genome sequence clades were defined as having boot-strap confidence value of 99 or higher [41], these clades were independently confirmed using principal component analysis. Clades were named using the corresponding MIRU-VNTR and spoligotype independently by the Institut Pasteur Guadeloupe according to published protocols (www.miru-vntrplus.org and www.pasteur-guadeloupe.fr:8081/SITVIT_ONLINE) [42]. Ancestral state reconstruction was undertaken using maximum parsimony, likelihood and Bayesian approaches.

### Identification and correlation of homoplastic variants

Homoplastic variants were identified by two techniques; 1) a Sanger in-house software that applied the accelerated transformation algorithm to a maximum parsimony tree and 2) phyC software [7] that pre-specified drug resistant and drug susceptible states and compared the occurrence of independent ancestral changes between the two groups. Both methods counted the number of independent occasions in which an ancestral base was different to the descendent base at any given site in the tree. The phyC method also calculated the Fisher’s exact statistic for the comparison of the number of homoplastic events that occurred in drug susceptible versus drug resistant strains. Given that some of the ESX genes are paralogous we also visually inspected the SNP calls within ESX genes to ensure that the mapping was accurate and coverage reliable enough to call the SNP. Correlation between homoplastic variant sites was undertaken in R using the cor.table function from the picante package.

### Principal component analysis genome wide association

Genome wide analysis with Bonferroni correction and correction for underlying genetic structure by principal components was performed for the following variables; resistance to first and second line drugs (kanamycin, ciprofloxacin, capreomycin), sputum smear status, gender, HIV status and previous treatment history using the function dapc from the package adegenet [43,44].

### Comparing whole genome, MIRU, MIRU-spoligotpye dendrograms

The correlation between whole genome, MIRU, MIRU-Spoligotype derived dendrograms was determined using the R program “Dendextend”. Co-phenetic correlations were obtained as per the Dendextend reference manual [45].

### Determining polymorphisms most disruptive of phylogenetic structure

Kendall et al [46,47] proposed a tree comparison method for determining variant sites most influential of tree structure in which a "reference" tree is constructed from the whole alignment, then compared to “experimental” trees built with the variant site in question removed. We used this method to detect phylogenetically informative and therefore potentially biologically informative polymorphisms in our alignment. This method will detect Homoplasy as does phyC and the Sanger software cited above, however it will also identify single ancestral sites that have particular influence on tree structure.

We noted that in a random sample of 100 single sites that belonged to blocks of 10 adjacent alignment sites which when removed did not cause a change in the initial screening, none were found to cause a change in the tree. It is widely accepted that tuberculosis does not undergo recombination, and in the absence of recombination, under circumstances with sufficient genetic diversity to recreate high-quality phylogenetic trees, removal of a single variable site from an alignment of ~20,000 variable sites should not affect the topology of the reconstructed phylogeny. While the information in all variable sites is pooled to reconstruct the phylogeny, we term sites whose removal led to an altered tree topology “phylogenetically disruptive”.

Using RAxML 7.2.4 [40] with the GTRCAT model of rate heterogeneity [48] to construct our trees, we created a reference tree from all 20976 variant sites. The same settings were then used to create trees from the alignment with the site in question removed. For an initial screening and to minimize computational time we removed blocks of ten sites from the alignment at a time. We compared the trees as per Kendall et al [46], using function refTreeDist from R package treescape [49]. We found that around 10% of the blocks of 10 variant sites, when removed, produced a tree with a different topology from the reference tree. We then re-ran the method, removing a single site of the alignment at a time, each belonging to a block of 10 that caused a change in the first run.

## Supporting information

S1 Fig The cophenetic correlation coefficient of whole genome sequence, MIRU, MIRU and spoligotype combined (MIRU Spol) and spoligotype dendrograms. (EPS)

S2 Fig Hotspots of correlation between homoplastic sites. White and yellow indicate significant positive correlation (p<0.05) while red indicates significant negative correlation (p<0.05).
(EPS)

S3 Fig Genome wide analysis with correction for population structure by principle components and Bonferroni correction for rifampicin, isoniazid, pyrazinamide and ethambutol drug resistance polymorphisms in *M*. *tuberculosis*. (EPS)

S4 Fig Genome wide analysis with correction for population structure by principle components and Bonferroni correction for streptomycin, kanamycin, capreomycin and ciprofloxacin drug resistance polymorphisms in *M*. *tuberculosis*. (EPS)

S1 Table Study metadata. (XLSX)

S2 Table The most homoplastic polymorphisms. (DOCX)

S3 Table Homoplastic polymorphisms detected by phyC. (DOCX)

S4 Table The most topologically influential polymorphisms. (DOCX)

S5 Table Accession numbers for genomes sequenced. (DOCX)
