# Supplementary material for: Convergent evolution and topologically disruptive polymorphisms among multidrug-resistant tuberculosis in Peru
Source: PLoS One. 2017 Dec 27;12(12):e0189838. doi: 10.1371/journal.pone.0189838 (PMC5744980; doi:10.1371/journal.pone.0189838)
Supplement: S2 Table — (DOCX) [file pone.0189838.s006.docx]

Supplementary Table S1

| Reference ID | Gene Name | Number  Homoplastic  Sites | Mutation | Mutation Type | n  Strains | Function Note |
| --- | --- | --- | --- | --- | --- | --- |
| 2155176 | katG | 21 | G->C | Ser315Thr | 340 | DR |
| 761160 | rpoB | 20 | C->T | Ser450Leu | 223 | DR |
| 761115 | rpoB | 15 | A->T | Asp435Val | 76 | DR |
| 1473254 | rrs | 15 | A->G | -  Position 1400 | 34 | DR |
| 4247436 | embB | 15 | A->G | Met306Val | 46 | DR |
| 1673433 | Int | 13 | C->T | - | 64 | DR |
| 2123153 | lldD2 | 12 | C->T | Val3Ile | 70 | Possible Anaerobic Adaptation |
| 3135920 | Rv2828 | 12 | G->C | Thr141Arg | 136 | Possible Toxin/Anti-Toxin |
| 4247438 | embB | 12 | G->A | Met306Ile | 50 | DR |
| 1340675 | esxK | 9 | A->G | S (Codon 3) | 84 | Immunogenic |
| 761144 | rpoB | 8 | C->T | His445Tyr | 15 | DR |
| 3136343 | Intergenic | 8 | G->A | - | 192 | 2bp upstream of Rv2828 |
| 3820553 | Intergenic | 7 | A->G | - | 214 | Unknown |
| 761144 | rpoB | 6 | C->G | His445Asp | 9 | DR |
| 781692 | rpsL | 6 | A->G | Lys43Arg | 36 | DR |
| 2122403 | lldD2 | 6 | C->T | Val253Met | 83 | Possible Anaerobic Adaptation |
| 2945175 | Intergenic | 6 | G->T | - | 244 | Unknown |
| 4247438 | embB | 6 | G->C | Met306Ile | 10 | DR |
| 4247581 | embB | 6 | A->C | Asp354Ala | 19 | DR |
| 7582 | gyrA | 5 | A->G | Asp94Ala | 10 | DR |
| 764822 | rpoC | 5 | T->G | Val483Ala | 11 | DR |
| 1094544 | Int | 5 | T->G | - | 10 | Unknown |
| 4247737 | embB | 5 | G->A | Gly406Asp | 16 | DR |
| 4249590 | embB | 5 | G->A | Asp1024Asn | 7 | DR |
| 7570 | gyrA | 4 | C->T | Ala90Val | 9 | DR |
| 761166 | rpoB | 4 | T->C | Leu452Pro | 5 | DR |
| 764822 | rpoC | 4 | T->C | Val483Ala | 6 | DR |
| 2338999 | Rv2082 | 4 | G->C | S (codon 94) | 121 | Unknown |
| 2339003 | Rv2082 | 4 | G->A | Ala96Thr | 121 | Unknown |
| 3122583 | Int | 4 | G->C | - | 147 | Unknown |
| 3232711 | Int | 4 | G->A | - | 53 | Unknown |
| 3594408 | Int | 4 | A->G | - | 32 | Unknown |
| 4247737 | embB | 4 | G->C | Gly406Asp | 20 | DR |
| 1160796 | esxI | 3 | G->A | S | 390 | Immunogenic |
| 1341052 | esxL | 3 | C->T | S | 49 | Immunogenic |
| 1674056 | fabG1 | 3 | G->A | S | 6 | DR |
| 2074522 | Intergenic | 3 | G->C | - | 121 | Unknown |
| 2123190 | Rv1873 | 3 | A->C | S | 100 | Unknown |
| 2289261 | Intergenic | 3 | T->C | - | 5 | Upstream of pncA (pyrazinamide resistance) |
| 2302042 | pks12 | 3 | G->A | NS | 132 | Virulence |
| 2338970 | Rv2082 | 3 | G->A | NS | 43 | Unknown |
| 2626686 | Rv2348 | 3 | A->C | NS | 15 | Adjacent to ESX genes |
| 3067969 | Intergenic | 3 | G->A | - | 19 | DR Upstream gene thyX (resistance to para-aminosalicylic acid) |
| 3594402 | Intergenic | 3 | G->C | - | 31 | Unknown |
| 3594403 | Intergenic | 3 | G->A | - | 31 | Unknown |
| 3594406 | Intergenic | 3 | T->C | - | 31 | Unknown |
| 4247736 | embB | 3 | G->A | NS | 6 | DR |
| 332761 | Rv0277 | 2 | A->G | S | 3 | Toxin Antitoxin |
| 761114 | rpoB | 2 | G->T | NS | 8 | DR |
| 761282 | rpoB | 2 | A->T | NS | 4 | DR |
| 764953 | rpoC | 2 | T->G | NS | 3 | DR |
| 766493 | rpoC | 2 | C->G | NS | 4 | DR |
| 1160767 | esxI | 2 | G->A | NS | 394 | Immunogenic |
| 1340665 | Intergenic | 2 | A->G | - | 57 | 2bp Upstream of esxK |
| 1341128 | esxL | 2 | A->G | NS | 68 | Immunogenic |
| 1472367 | rrs | 2 | A->C | NS | 5 | DR |
| 2030363 | esxM | 2 | A->G | S | 22 | Immunogenic |
| 2030950 | esxN | 2 | G->A | S | 30 | Immunogenic |
| 2155175 | katG | 2 | G->T | S | 8 | DR |
| 2296190 | pks12 | 2 | A->G | S | 114 | Virulence |
| 2338819 | Rv2082 | 2 | T->C | S | 49 | Unknown |
| 2338820 | Rv2082 | 2 | A->G | NS | 49 | Unknown |
| 2401892 | Intergenic | 2 | C->T | - | 245 | Unknown |
| 2532184 | Intergenic | 2 | C->G | - | 8 | Unknown |
| 2626103 | esxO | 2 | C->G | S | 18 | Immunogenic |
| 2626608 | Intergenic | 2 | G->A | - | 21 | Upstream of esxP |
| 2867355 | lppB | 2 | A->G | NS | 150 | Lipoprotein |
| 2867583 | lppB | 2 | T->C | NS | 24 | Lipoprotein |
| 2867602 | lppB | 2 | G->A | S | 24 | Lipoprotein |
| 2965908 | Intergenic | 2 | C->T | - | 3 | Unknown |
| 2986835 | Rv2670 | 2 | G->A | NS | 4 | DR |
| 3074503 | Intergenic | 2 | G->A | - | 19 | Upstream of thyA (associated with PAS resistance) |
| 3336536 | Intergenic | 2 | T->A | - | 10 | Unknown |
| 3343419 | hupB | 2 | C->T | NS | 17 | DNA Binding |
| 3691011 | Intergenic | 2 | C->A | - | 19 | Unknown |
| 3691013 | Intergenic | 2 | A->G | - | 19 | Unknown |
| 3841660 | Intergenic | 2 | T->A | - | 40 | Unknown |
| 3841662 | Intergenic | 2 | T->G | - | 45 | Unknown |
| 3841670 | Intergenic | 2 | T->C | - | 77 | Unknown |
| 3841671 | Intergenic | 2 | C->T | - | 68 | Unknown |
| 3877967 | rpoA | 2 | A->C | NS | 3 | DR or  Compensation |
| 4060108 | esxV | 2 | G->A | NS | 391 | Immunogenic |
| 4243228 | Intergenic | 2 | C->T | - | 3 | Upstream of embA (Ethambutol Resistance) |
