# Supplementary material for: Convergent evolution and topologically disruptive polymorphisms among multidrug-resistant tuberculosis in Peru
Source: PLoS One. 2017 Dec 27;12(12):e0189838. doi: 10.1371/journal.pone.0189838 (PMC5744980; doi:10.1371/journal.pone.0189838)
Supplement: S3 Table — (DOCX) [file pone.0189838.s007.docx]

Supplementary Table S2

| Name | Reference position | p-value |
| --- | --- | --- |
| rpoB | 761160 | 0.00029 |
| katG | 2155176 | 0.00057 |
| rpoB | 761144 | 0.00063 |
| embB | 4247436 | 0.00063 |
| embB | 4247438 | 0.00099 |
| rpoB | 761115 | 0.00148 |
| rrs | 1473254 | 0.00148 |
| gyrA | 7582 | 0.00206 |
| rpoB | 761145 | 0.00339 |
| rpoC | 764822 | 0.00339 |
| embB | 4247581 | 0.00339 |
| embB | 4247737 | 0.00339 |
| gyrA | 7570 | 0.00503 |
| rpoC | 766493 | 0.00503 |
| embB | 4249590 | 0.00503 |
| lldD2 | 2123153 | 0.00598 |
| rpoC | 767128 | 0.00714 |
| cysA2 | 909285 | 0.01095 |
| rpoA | 3877967 | 0.01095 |
| Rv2828c | 3135920 | 0.01369 |
| esxK | 1340675 | 0.01556 |
| gyrA | 7581 | 0.03741 |
| bglS | 217682 | 0.03741 |
| Rv0277c | 332918 | 0.03741 |
| rpoB | 761161 | 0.03741 |
| rpoC | 765468 | 0.03741 |
| Rv1140 | 1268028 | 0.03741 |
| rrs | 1472759 | 0.03741 |
| Rv1435c | 1612603 | 0.03741 |
| Rv1610 | 1809650 | 0.03741 |
| esxN | 2030950 | 0.03741 |
| pncA | 2288736 | 0.03741 |
| pncA | 2289216 | 0.03741 |
| pncA | 2288739 | 0.03741 |
| pncA | 2288827 | 0.03741 |
| pncA | 2288943 | 0.03741 |
| pncA | 2289039 | 0.03741 |
| pks12 | 2302042 | 0.03741 |
| pks12 | 2306315 | 0.03741 |
| hisE | 2380761 | 0.03741 |
| Rv2348c | 2626686 | 0.03741 |
| Rv2571c | 2895326 | 0.03741 |
| Rv2670c | 2986835 | 0.03741 |
| nuoI | 3520985 | 0.03741 |
| PS00583 pfkB | 3935454 | 0.03741 |
| esxV | 4060238 | 0.03741 |
| embB | 4247736 | 0.03741 |
| ethA | 4327449 | 0.03741 |
| ethA | 4326720 | 0.03741 |
