# Supplementary material for: Convergent evolution and topologically disruptive polymorphisms among multidrug-resistant tuberculosis in Peru
Source: PLoS One. 2017 Dec 27;12(12):e0189838. doi: 10.1371/journal.pone.0189838 (PMC5744980; doi:10.1371/journal.pone.0189838)
Supplement: S4 Table — (DOCX) [file pone.0189838.s008.docx]

Supplementary Table S3

| Name | Reference Position | Distance Between Trees (Before and After Removal) | Mutation | Mutation Type | Function Note |
| --- | --- | --- | --- | --- | --- |
| esxI | 1160776 | 239.91 | T->A | NS | Immunogenic |
| Rv1319c | 1480980 | 239.90 | T->C | S | Catalyses synthesis of CAMP |
| Rv3113 | 3480482 | 186.95 | G->A | NS | Unknown |
| Intergenic | 3841670 | 180.45 | T->C | S | Intergenic |
| rpoB | 761160 | 160.41 | C->G | NS | DR |
| Rv0731c | 824367 | 158.57 | T->C | NS | Possible Methyltransferase |
| Intergenic | 1096639 | 143.13 | T->G | S | Intergenic |
| pks12 | 2302042 | 125.85 | G->A | NS | Virulence |
| Rv0276 | 332150 | 120.63 | A->G | NS | Unknown next to VapC25 toxin |
| pks12 | 2300675 | 114.25 | G->A | S | Virulence |
| pks12 | 2300678 | 114.25 | G->C | S | Virulence |
| gid | 4407979 | 106.05 | A->C | NS | Glucose inhibited division protein |
| pks12 | 2302280 | 87.63 | A->G | S | Virulence |
| katG | 2155025 | 73.81 | C->A | S | DR |
| cydB | 1823557 | 72.25 | G->T | NS | Respiratory chain terminal step |
| katG | 2155176 | 65.02 | C->A | S | DR |
| rpoB | 761115 | 61.48 | A->T | NS | DR |
| esxV | 4060108 | 55.59 | G->A | S | Immunogenic |
| embB | 4247438 | 44.79 | G->A | NS | DR |
| Intergenic | 2945175 | 44.54 | G->T | S | Intergenic |
| esxO | 2626103 | 42.54 | C->A | S | Immunogenic |
| Intergenic | 1094381 | 42.50 | C->G | S | Intergenic |
| Intergenic | 1673433 | 42.36 | C->T | S | Intergenic |
| Rv2828c | 3135920 | 42.36 | G->C | NS | Possible Toxin/Anti-Toxin |
| Rv1043c | 1166448 | 41.79 | G->C | NS | Unknown |
| cyst | 2695903 | 41.79 | G->A | S | Active transport of sulfur compounds |
| Intergenic | 3621427 | 41.79 | G->A | S | Intergenic |
| purH | 1069456 | 41.75 | T->G | NS | De Novo Purine Synthesis |
| esxL | 1341040 | 39.82 | T->C | S | Immunogenic |
| Intergenic | 1313554 | 38.07 | G->A | S | Intergenic |
| Intergenic | 4120990 | 34.97 | A->G | S | Intergenic |
| Rv2525c | 2850349 | 32.63 | G->A | S | Possible cell wall biosynthesis |
| Rv0502 | 593653 | 32.45 | G->A | S | Unknown |
| esxL | 1341182 | 32.33 | T->C | S | Immunogenic |
| Nrp | 117020 | 32.09 | A->C | NS | Lipid Metabolism |
| Rv0111 | 135802 | 32.09 | A->G | NS | Unknown |
| Intergenic | 144024 | 32.09 | C->A | S | Intergenic |
| Intergenic | 336346 | 32.09 | A->G | S | Intergenic |
| Intergenic | 336347 | 32.09 | T->C | S | Intergenic |
| Intergenic | 336355 | 32.09 | C->T | S | Intergenic |
| Intergenic | 547580 | 32.09 | G->T | S | Intergenic |
| Rv0541c | 634046 | 32.09 | C->G | NS | Unknown |
| Rv0695 | 794920 | 32.09 | G->C | NS | Unknown |
| Rv0825c | 919426 | 32.09 | G->C | NS | Unknown |
| Rv0892 | 995119 | 32.09 | A->G | NS | Unknown |
| Intergenic | 999370 | 32.09 | G->C | S | Intergenic |
| pstS1 | 1043061 | 32.09 | A->G | NS | Active transport of phosphate across the membrane |
| mprB | 1099005 | 32.09 | T->G | NS | Two component regulatory system |
| trcS | 1157555 | 32.09 | G->C | NS | Two component regulatory system |
| lipU | 1201296 | 32.09 | G->A | NS | Hydrolyses lipids |
| Rv1081c | 1206407 | 32.09 | G->A | S | Unknown |
| Intergenic | 1267260 | 32.09 | G->A | S | Intergenic |
| esxL | 1341081 | 32.09 | T->C | NS | Immunogenic |
