# Supplementary material for: Convergent evolution and topologically disruptive polymorphisms among multidrug-resistant tuberculosis in Peru
Source: PLoS One. 2017 Dec 27;12(12):e0189838. doi: 10.1371/journal.pone.0189838 (PMC5744980; doi:10.1371/journal.pone.0189838)
Supplement: S5 Table — (DOCX) [file pone.0189838.s009.docx]

Supplementary Table S4

| ENAProjectAccesion | ProjectAccesion | SampleAccession | SecondarySampleAccession | ContigAccession | LaneName | CollectionYear |
| --- | --- | --- | --- | --- | --- | --- |
| ERP004677 | PRJEB5280 | ERZ369326 | ERS518733 | FPNV01000001-FPNV01000062 | 14722_6#1 | 2008 |
| ERP004677 | PRJEB5280 | ERZ369327 | ERS518761 | FPNA01000001-FPNA01000076 | 14722_6#10 | 2009 |
| ERP004677 | PRJEB5280 | ERZ369328 | ERS518764 | FPNH01000001-FPNH01000074 | 14722_6#11 | 2008 |
| ERP004677 | PRJEB5280 | ERZ369329 | ERS518765 | FPOK01000001-FPOK01000089 | 14722_6#12 | 2009 |
| ERP004677 | PRJEB5280 | ERZ369330 | ERS518766 | FPNK01000001-FPNK01000060 | 14722_6#13 | 2009 |
| ERP004677 | PRJEB5280 | ERZ369331 | ERS518767 | FPNL01000001-FPNL01000065 | 14722_6#14 | 2009 |
| ERP004677 | PRJEB5280 | ERZ369332 | ERS518769 | FPNN01000001-FPNN01000060 | 14722_6#15 | 2008 |
| ERP004677 | PRJEB5280 | ERZ369333 | ERS518771 | FPNT01000001-FPNT01000060 | 14722_6#16 | 2008 |
| ERP004677 | PRJEB5280 | ERZ369334 | ERS518772 | FPNZ01000001-FPNZ01000060 | 14722_6#17 | 2008 |
| ERP004677 | PRJEB5280 | ERZ369335 | ERS518784 | FPOL01000001-FPOL01000071 | 14722_6#18 | 2009 |
| ERP004677 | PRJEB5280 | ERZ369336 | ERS518786 | FPMX01000001-FPMX01000062 | 14722_6#19 | 2009 |
| ERP004677 | PRJEB5280 | ERZ369337 | ERS518737 | FPNW01000001-FPNW01000068 | 14722_6#2 | 2009 |
| ERP004677 | PRJEB5280 | ERZ369338 | ERS518787 | FPOJ01000001-FPOJ01000061 | 14722_6#20 | 2008 |
| ERP004677 | PRJEB5280 | ERZ369339 | ERS518789 | FPNY01000001-FPNY01000078 | 14722_6#21 | 2009 |
| ERP004677 | PRJEB5280 | ERZ369340 | ERS518791 | FPMY01000001-FPMY01000060 | 14722_6#22 | 2009 |
| ERP004677 | PRJEB5280 | ERZ369341 | ERS518796 | FPNJ01000001-FPNJ01000062 | 14722_6#23 | 2009 |
| ERP004677 | PRJEB5280 | ERZ369342 | ERS518800 | FPMW01000001-FPMW01000065 | 14722_6#24 | 2009 |
| ERP004677 | PRJEB5280 | ERZ369343 | ERS518805 | FPOD01000001-FPOD01000065 | 14722_6#25 | 2009 |
| ERP004677 | PRJEB5280 | ERZ369344 | ERS518808 | FQNN01000001-FQNN01000076 | 14722_6#26 | 2009 |
| ERP004677 | PRJEB5280 | ERZ369345 | ERS518819 | FPNG01000001-FPNG01000079 | 14722_6#27 | 2009 |
| ERP004677 | PRJEB5280 | ERZ369346 | ERS518822 | FPOI01000001-FPOI01000071 | 14722_6#28 | 2009 |
| ERP004677 | PRJEB5280 | ERZ369347 | ERS518823 | FPNP01000001-FPNP01000079 | 14722_6#29 | 2009 |
| ERP004677 | PRJEB5280 | ERZ369348 | ERS518742 | FPOC01000001-FPOC01000058 | 14722_6#3 | 2009 |
| ERP004677 | PRJEB5280 | ERZ369349 | ERS518828 | FPNM01000001-FPNM01000056 | 14722_6#30 | 2009 |
| ERP004677 | PRJEB5280 | ERZ369350 | ERS518829 | FPNU01000001-FPNU01000062 | 14722_6#31 | 2009 |
| ERP004677 | PRJEB5280 | ERZ369351 | ERS518830 | FPOA01000001-FPOA01000072 | 14722_6#32 | 2009 |
| ERP004677 | PRJEB5280 | ERZ369352 | ERS518831 | FPOG01000001-FPOG01000070 | 14722_6#33 | 2009 |
| ERP004677 | PRJEB5280 | ERZ369353 | ERS518832 | FPMZ01000001-FPMZ01000064 | 14722_6#34 | 2009 |
| ERP004677 | PRJEB5280 | ERZ369354 | ERS518833 | FPNI01000001-FPNI01000069 | 14722_6#35 | 2009 |
| ERP004677 | PRJEB5280 | ERZ369355 | ERS518836 | FPNS01000001-FPNS01000083 | 14722_6#36 | 2009 |
| ERP004677 | PRJEB5280 | ERZ369356 | ERS518837 | FPNC01000001-FPNC01000067 | 14722_6#37 | 2009 |
| ERP004677 | PRJEB5280 | ERZ369357 | ERS518838 | FPOE01000001-FPOE01000064 | 14722_6#38 | 2009 |
| ERP004677 | PRJEB5280 | ERZ369358 | ERS518839 | FPON01000001-FPON01000073 | 14722_6#39 | 2009 |
| ERP004677 | PRJEB5280 | ERZ369359 | ERS518744 | FPNX01000001-FPNX01000067 | 14722_6#4 | 2009 |
| ERP004677 | PRJEB5280 | ERZ369360 | ERS518840 | FPNB01000001-FPNB01000071 | 14722_6#40 | 2009 |
| ERP004677 | PRJEB5280 | ERZ369361 | ERS518842 | FPOH01000001-FPOH01000082 | 14722_6#41 | 2009 |
| ERP004677 | PRJEB5280 | ERZ369362 | ERS518845 | FPND01000001-FPND01000067 | 14722_6#42 | 2009 |
| ERP004677 | PRJEB5280 | ERZ369363 | ERS518846 | FPNQ01000001-FPNQ01000088 | 14722_6#43 | 2009 |
| ERP004677 | PRJEB5280 | ERZ369364 | ERS518851 | FPOF01000001-FPOF01000067 | 14722_6#44 | 2009 |
| ERP004677 | PRJEB5280 | ERZ369365 | ERS518855 | FPOM01000001-FPOM01000067 | 14722_6#45 | 2009 |
| ERP004677 | PRJEB5280 | ERZ369366 | ERS518856 | FPNE01000001-FPNE01000086 | 14722_6#46 | 2009 |
| ERP004677 | PRJEB5280 | ERZ369367 | ERS518857 | FPNO01000001-FPNO01000078 | 14722_6#47 | 2009 |
| ERP004677 | PRJEB5280 | ERZ369368 | ERS518858 | FPNF01000001-FPNF01000066 | 14722_6#48 | 2009 |
| ERP004677 | PRJEB5280 | ERZ369369 | ERS518859 | FPOO01000001-FPOO01000066 | 14722_6#49 | 2009 |
| ERP004677 | PRJEB5280 | ERZ369370 | ERS518747 | FPNR01000001-FPNR01000067 | 14722_6#5 | 2009 |
| ERP004677 | PRJEB5280 | ERZ369371 | ERS518860 | FPOB01000001-FPOB01000069 | 14722_6#50 | 2009 |
| ERP004677 | PRJEB5280 | ERZ369372 | ERS518863 | FQOL01000001-FQOL01000067 | 14722_6#51 | 2009 |
| ERP004677 | PRJEB5280 | ERZ369373 | ERS518864 | FPOP01000001-FPOP01000070 | 14722_6#52 | 2009 |
| ERP004677 | PRJEB5280 | ERZ369374 | ERS518867 | FPOT01000001-FPOT01000076 | 14722_6#53 | 2009 |
| ERP004677 | PRJEB5280 | ERZ369375 | ERS518873 | FPOQ01000001-FPOQ01000071 | 14722_6#54 | 2009 |
| ERP004677 | PRJEB5280 | ERZ369376 | ERS518874 | FPOR01000001-FPOR01000069 | 14722_6#55 | 2009 |
| ERP004677 | PRJEB5280 | ERZ369377 | ERS518877 | FQNI01000001-FQNI01000071 | 14722_6#56 | 2009 |
| ERP004677 | PRJEB5280 | ERZ369378 | ERS518878 | FPOU01000001-FPOU01000066 | 14722_6#57 | 2009 |
| ERP004677 | PRJEB5280 | ERZ369379 | ERS518880 | FPOS01000001-FPOS01000066 | 14722_6#58 | 2009 |
| ERP004677 | PRJEB5280 | ERZ369380 | ERS518881 | FPOW01000001-FPOW01000071 | 14722_6#59 | 2009 |
| ERP004677 | PRJEB5280 | ERZ369381 | ERS518749 | FPOV01000001-FPOV01000070 | 14722_6#6 | 2009 |
| ERP004677 | PRJEB5280 | ERZ369382 | ERS518884 | FPOY01000001-FPOY01000082 | 14722_6#60 | 2009 |
| ERP004677 | PRJEB5280 | ERZ369383 | ERS518885 | FPOX01000001-FPOX01000062 | 14722_6#61 | 2009 |
| ERP004677 | PRJEB5280 | ERZ369384 | ERS518888 | FPPB01000001-FPPB01000070 | 14722_6#62 | 2009 |
| ERP004677 | PRJEB5280 | ERZ369385 | ERS518894 | FPPA01000001-FPPA01000072 | 14722_6#63 | 2009 |
| ERP004677 | PRJEB5280 | ERZ369386 | ERS518895 | FPOZ01000001-FPOZ01000060 | 14722_6#64 | 2009 |
| ERP004677 | PRJEB5280 | ERZ369387 | ERS518896 | FPPU01000001-FPPU01000167 | 14722_6#65 | 2009 |
| ERP004677 | PRJEB5280 | ERZ369388 | ERS518897 | FPPI01000001-FPPI01000070 | 14722_6#66 | 2009 |
| ERP004677 | PRJEB5280 | ERZ369389 | ERS518899 | FPPF01000001-FPPF01000077 | 14722_6#67 | 2009 |
| ERP004677 | PRJEB5280 | ERZ369390 | ERS518902 | FPPC01000001-FPPC01000070 | 14722_6#68 | 2009 |
| ERP004677 | PRJEB5280 | ERZ369391 | ERS518903 | FPPG01000001-FPPG01000072 | 14722_6#69 | 2009 |
| ERP004677 | PRJEB5280 | ERZ369392 | ERS518750 | FPPH01000001-FPPH01000073 | 14722_6#7 | 2009 |
| ERP004677 | PRJEB5280 | ERZ369393 | ERS518904 | FPPS01000001-FPPS01000076 | 14722_6#70 | 2009 |
| ERP004677 | PRJEB5280 | ERZ369394 | ERS518905 | FPPO01000001-FPPO01000072 | 14722_6#71 | 2009 |
| ERP004677 | PRJEB5280 | ERZ369395 | ERS518906 | FPPQ01000001-FPPQ01000066 | 14722_6#72 | 2009 |
| ERP004677 | PRJEB5280 | ERZ369396 | ERS518907 | FPPD01000001-FPPD01000069 | 14722_6#73 | 2009 |
| ERP004677 | PRJEB5280 | ERZ369397 | ERS518910 | FPPM01000001-FPPM01000068 | 14722_6#74 | 2009 |
| ERP004677 | PRJEB5280 | ERZ369398 | ERS518912 | FPPK01000001-FPPK01000075 | 14722_6#75 | 2009 |
| ERP004677 | PRJEB5280 | ERZ369399 | ERS518913 | FPPN01000001-FPPN01000066 | 14722_6#76 | 2009 |
| ERP004677 | PRJEB5280 | ERZ369400 | ERS518914 | FPPP01000001-FPPP01000073 | 14722_6#77 | 2009 |
| ERP004677 | PRJEB5280 | ERZ369401 | ERS518915 | FPPE01000001-FPPE01000075 | 14722_6#78 | 2009 |
| ERP004677 | PRJEB5280 | ERZ369402 | ERS518917 | FPPL01000001-FPPL01000074 | 14722_6#79 | 2009 |
| ERP004677 | PRJEB5280 | ERZ369403 | ERS518751 | FPPJ01000001-FPPJ01000080 | 14722_6#8 | 2009 |
| ERP004677 | PRJEB5280 | ERZ369404 | ERS518918 | FPPR01000001-FPPR01000066 | 14722_6#80 | 2009 |
| ERP004677 | PRJEB5280 | ERZ369405 | ERS518919 | FPPT01000001-FPPT01000083 | 14722_6#81 | 2010 |
| ERP004677 | PRJEB5280 | ERZ369406 | ERS518920 | FPPV01000001-FPPV01000072 | 14722_6#82 | 2009 |
| ERP004677 | PRJEB5280 | ERZ369407 | ERS518921 | FPPX01000001-FPPX01000065 | 14722_6#83 | 2010 |
| ERP004677 | PRJEB5280 | ERZ369408 | ERS518922 | FPPW01000001-FPPW01000067 | 14722_6#84 | 2009 |
| ERP004677 | PRJEB5280 | ERZ369409 | ERS518923 | FPPY01000001-FPPY01000068 | 14722_6#85 | 2010 |
| ERP004677 | PRJEB5280 | ERZ369410 | ERS518924 | FPPZ01000001-FPPZ01000072 | 14722_6#86 | 2009 |
| ERP004677 | PRJEB5280 | ERZ369411 | ERS518926 | FPQA01000001-FPQA01000059 | 14722_6#87 | 2009 |
| ERP004677 | PRJEB5280 | ERZ369412 | ERS518927 | FPQC01000001-FPQC01000075 | 14722_6#88 | 2009 |
| ERP004677 | PRJEB5280 | ERZ369413 | ERS518928 | FPQB01000001-FPQB01000066 | 14722_6#89 | 2009 |
| ERP004677 | PRJEB5280 | ERZ369414 | ERS518760 | FPQD01000001-FPQD01000068 | 14722_6#9 | 2008 |
| ERP004677 | PRJEB5280 | ERZ369415 | ERS518929 | FPQE01000001-FPQE01000065 | 14722_6#90 | 2009 |
| ERP004677 | PRJEB5280 | ERZ369416 | ERS518931 | FPQF01000001-FPQF01000069 | 14722_6#91 | 2009 |
| ERP004677 | PRJEB5280 | ERZ369417 | ERS518932 | FPQG01000001-FPQG01000074 | 14722_6#92 | 2009 |
| ERP004677 | PRJEB5280 | ERZ369418 | ERS518933 | FPQH01000001-FPQH01000081 | 14722_6#93 | 2009 |
| ERP004677 | PRJEB5280 | ERZ369419 | ERS518934 | FPQI01000001-FPQI01000067 | 14722_6#94 | 2009 |
| ERP004677 | PRJEB5280 | ERZ369420 | ERS518935 | FPQJ01000001-FPQJ01000078 | 14722_6#95 | 2009 |
| ERP004677 | PRJEB5280 | ERZ369421 | ERS519134 | FPQK01000001-FPQK01000077 | 14722_7#1 | 2011 |
| ERP004677 | PRJEB5280 | ERZ369422 | ERS519177 | FPQL01000001-FPQL01000078 | 14722_7#10 | 2012 |
| ERP004677 | PRJEB5280 | ERZ369423 | ERS519178 | FPQM01000001-FPQM01000076 | 14722_7#11 | 2012 |
| ERP004677 | PRJEB5280 | ERZ369424 | ERS519179 | FPQP01000001-FPQP01000078 | 14722_7#12 | 2012 |
| ERP004677 | PRJEB5280 | ERZ369425 | ERS519182 | FPQN01000001-FPQN01000069 | 14722_7#13 | 2012 |
| ERP004677 | PRJEB5280 | ERZ369426 | ERS519184 | FPQQ01000001-FPQQ01000081 | 14722_7#14 | 2012 |
| ERP004677 | PRJEB5280 | ERZ369427 | ERS519185 | FPQO01000001-FPQO01000073 | 14722_7#15 | 2012 |
| ERP004677 | PRJEB5280 | ERZ369428 | ERS519187 | FPQR01000001-FPQR01000086 | 14722_7#16 | 2012 |
| ERP004677 | PRJEB5280 | ERZ369429 | ERS519188 | FPQS01000001-FPQS01000085 | 14722_7#17 | 2012 |
| ERP004677 | PRJEB5280 | ERZ369430 | ERS519189 | FQNP01000001-FQNP01000074 | 14722_7#18 | 2012 |
| ERP004677 | PRJEB5280 | ERZ369431 | ERS519193 | FPQT01000001-FPQT01000086 | 14722_7#19 | 2012 |
| ERP004677 | PRJEB5280 | ERZ369432 | ERS519135 | FPQU01000001-FPQU01000068 | 14722_7#2 | 2011 |
| ERP004677 | PRJEB5280 | ERZ369433 | ERS519194 | FPQX01000001-FPQX01000082 | 14722_7#20 | 2012 |
| ERP004677 | PRJEB5280 | ERZ369434 | ERS519195 | FQNL01000001-FQNL01000069 | 14722_7#21 | 2012 |
| ERP004677 | PRJEB5280 | ERZ369435 | ERS519196 | FPQV01000001-FPQV01000070 | 14722_7#22 | 2012 |
| ERP004677 | PRJEB5280 | ERZ369436 | ERS519197 | FPQW01000001-FPQW01000074 | 14722_7#23 | 2012 |
| ERP004677 | PRJEB5280 | ERZ369437 | ERS519198 | FPQY01000001-FPQY01000079 | 14722_7#24 | 2012 |
| ERP004677 | PRJEB5280 | ERZ369438 | ERS519199 | FPRE01000001-FPRE01000080 | 14722_7#25 | 2012 |
| ERP004677 | PRJEB5280 | ERZ369439 | ERS519204 | FPQZ01000001-FPQZ01000073 | 14722_7#26 | 2009 |
| ERP004677 | PRJEB5280 | ERZ369440 | ERS519206 | FPRC01000001-FPRC01000091 | 14722_7#27 | 2012 |
| ERP004677 | PRJEB5280 | ERZ369441 | ERS519209 | FQNQ01000001-FQNQ01000090 | 14722_7#28 | 2012 |
| ERP004677 | PRJEB5280 | ERZ369442 | ERS518734 | FPRD01000001-FPRD01000077 | 14722_7#29 | 2009 |
| ERP004677 | PRJEB5280 | ERZ369443 | ERS519141 | FPRA01000001-FPRA01000071 | 14722_7#3 | 2011 |
| ERP004677 | PRJEB5280 | ERZ369444 | ERS518735 | FPRB01000001-FPRB01000078 | 14722_7#30 | 2008 |
| ERP004677 | PRJEB5280 | ERZ369445 | ERS518736 | FPRF01000001-FPRF01000075 | 14722_7#31 | 2009 |
| ERP004677 | PRJEB5280 | ERZ369446 | ERS518738 | FPRI01000001-FPRI01000083 | 14722_7#32 | 2009 |
| ERP004677 | PRJEB5280 | ERZ369447 | ERS518739 | FPRM01000001-FPRM01000065 | 14722_7#33 | 2009 |
| ERP004677 | PRJEB5280 | ERZ369448 | ERS518740 | FPRL01000001-FPRL01000081 | 14722_7#34 | 2009 |
| ERP004677 | PRJEB5280 | ERZ369449 | ERS518741 | FPRH01000001-FPRH01000076 | 14722_7#35 | 2009 |
| ERP004677 | PRJEB5280 | ERZ369450 | ERS518743 | FPRG01000001-FPRG01000086 | 14722_7#36 | 2009 |
| ERP004677 | PRJEB5280 | ERZ369451 | ERS518745 | FPRJ01000001-FPRJ01000071 | 14722_7#37 | 2008 |
| ERP004677 | PRJEB5280 | ERZ369452 | ERS518746 | FPRK01000001-FPRK01000083 | 14722_7#38 | 2009 |
| ERP004677 | PRJEB5280 | ERZ369453 | ERS518748 | FPRO01000001-FPRO01000107 | 14722_7#39 | 2008 |
| ERP004677 | PRJEB5280 | ERZ369454 | ERS519146 | FPRN01000001-FPRN01000078 | 14722_7#4 | 2012 |
| ERP004677 | PRJEB5280 | ERZ369455 | ERS518752 | FPRP01000001-FPRP01000076 | 14722_7#40 | 2008 |
| ERP004677 | PRJEB5280 | ERZ369456 | ERS518753 | FPRQ01000001-FPRQ01000085 | 14722_7#41 | 2009 |
| ERP004677 | PRJEB5280 | ERZ369457 | ERS518754 | FPRR01000001-FPRR01000073 | 14722_7#42 | 2008 |
| ERP004677 | PRJEB5280 | ERZ369458 | ERS518755 | FPRS01000001-FPRS01000088 | 14722_7#43 | 2008 |
| ERP004677 | PRJEB5280 | ERZ369459 | ERS518756 | FPRT01000001-FPRT01000067 | 14722_7#44 | 2009 |
| ERP004677 | PRJEB5280 | ERZ369460 | ERS518757 | FPRU01000001-FPRU01000069 | 14722_7#45 | 2009 |
| ERP004677 | PRJEB5280 | ERZ369461 | ERS518758 | FQON01000001-FQON01000067 | 14722_7#46 | 2009 |
| ERP004677 | PRJEB5280 | ERZ369462 | ERS518759 | FPRV01000001-FPRV01000074 | 14722_7#47 | 2009 |
| ERP004677 | PRJEB5280 | ERZ369463 | ERS518762 | FPRW01000001-FPRW01000081 | 14722_7#48 | 2009 |
| ERP004677 | PRJEB5280 | ERZ369464 | ERS518763 | FPRX01000001-FPRX01000063 | 14722_7#49 | 2009 |
| ERP004677 | PRJEB5280 | ERZ369465 | ERS519149 | FPRZ01000001-FPRZ01000090 | 14722_7#5 | 2011 |
| ERP004677 | PRJEB5280 | ERZ369466 | ERS518768 | FPRY01000001-FPRY01000073 | 14722_7#50 | 2009 |
| ERP004677 | PRJEB5280 | ERZ369467 | ERS518770 | FPSA01000001-FPSA01000073 | 14722_7#51 | 2008 |
| ERP004677 | PRJEB5280 | ERZ369468 | ERS518773 | FPSB01000001-FPSB01000067 | 14722_7#52 | 2008 |
| ERP004677 | PRJEB5280 | ERZ369469 | ERS518774 | FPSC01000001-FPSC01000074 | 14722_7#53 | 2009 |
| ERP004677 | PRJEB5280 | ERZ369470 | ERS518775 | FPSD01000001-FPSD01000068 | 14722_7#54 | 2009 |
| ERP004677 | PRJEB5280 | ERZ369471 | ERS518776 | FPSE01000001-FPSE01000084 | 14722_7#55 | 2009 |
| ERP004677 | PRJEB5280 | ERZ369472 | ERS518777 | FPSH01000001-FPSH01000080 | 14722_7#56 | 2008 |
| ERP004677 | PRJEB5280 | ERZ369473 | ERS518778 | FPSF01000001-FPSF01000080 | 14722_7#57 | 2009 |
| ERP004677 | PRJEB5280 | ERZ369474 | ERS518779 | FPSG01000001-FPSG01000077 | 14722_7#58 | 2009 |
| ERP004677 | PRJEB5280 | ERZ369475 | ERS518780 | FPSI01000001-FPSI01000074 | 14722_7#59 | 2008 |
| ERP004677 | PRJEB5280 | ERZ369476 | ERS519158 | FQNH01000001-FQNH01000073 | 14722_7#6 | 2012 |
| ERP004677 | PRJEB5280 | ERZ369477 | ERS518781 | FPSK01000001-FPSK01000075 | 14722_7#60 | 2008 |
| ERP004677 | PRJEB5280 | ERZ369478 | ERS518782 | FPSJ01000001-FPSJ01000070 | 14722_7#61 | 2009 |
| ERP004677 | PRJEB5280 | ERZ369479 | ERS518783 | FPSL01000001-FPSL01000091 | 14722_7#62 | 2009 |
| ERP004677 | PRJEB5280 | ERZ369480 | ERS518785 | FQNK01000001-FQNK01000077 | 14722_7#63 | 2009 |
| ERP004677 | PRJEB5280 | ERZ369481 | ERS518788 | FPSN01000001-FPSN01000081 | 14722_7#64 | 2009 |
| ERP004677 | PRJEB5280 | ERZ369482 | ERS518790 | FPSM01000001-FPSM01000080 | 14722_7#65 | 2009 |
| ERP004677 | PRJEB5280 | ERZ369483 | ERS518792 | FPSO01000001-FPSO01000080 | 14722_7#66 | 2009 |
| ERP004677 | PRJEB5280 | ERZ369484 | ERS518793 | FPSP01000001-FPSP01000086 | 14722_7#67 | 2009 |
| ERP004677 | PRJEB5280 | ERZ369485 | ERS518794 | FPSQ01000001-FPSQ01000075 | 14722_7#68 | 2009 |
| ERP004677 | PRJEB5280 | ERZ369486 | ERS518795 | FQNU01000001-FQNU01000068 | 14722_7#69 | 2009 |
| ERP004677 | PRJEB5280 | ERZ369487 | ERS519168 | FPSR01000001-FPSR01000079 | 14722_7#7 | 2011 |
| ERP004677 | PRJEB5280 | ERZ369488 | ERS518797 | FPST01000001-FPST01000067 | 14722_7#70 | 2009 |
| ERP004677 | PRJEB5280 | ERZ369489 | ERS518798 | FPSS01000001-FPSS01000080 | 14722_7#71 | 2009 |
| ERP004677 | PRJEB5280 | ERZ369490 | ERS518799 | FPSU01000001-FPSU01000078 | 14722_7#72 | 2009 |
| ERP004677 | PRJEB5280 | ERZ369491 | ERS518801 | FPSW01000001-FPSW01000068 | 14722_7#73 | 2009 |
| ERP004677 | PRJEB5280 | ERZ369492 | ERS518802 | FPSV01000001-FPSV01000067 | 14722_7#74 | 2009 |
| ERP004677 | PRJEB5280 | ERZ369493 | ERS518803 | FPSX01000001-FPSX01000068 | 14722_7#75 | 2009 |
| ERP004677 | PRJEB5280 | ERZ369494 | ERS518804 | FPSY01000001-FPSY01000073 | 14722_7#76 | 2009 |
| ERP004677 | PRJEB5280 | ERZ369495 | ERS518806 | FPSZ01000001-FPSZ01000075 | 14722_7#77 | 2009 |
| ERP004677 | PRJEB5280 | ERZ369496 | ERS518807 | FPTA01000001-FPTA01000089 | 14722_7#78 | 2009 |
| ERP004677 | PRJEB5280 | ERZ369497 | ERS518809 | FPTB01000001-FPTB01000091 | 14722_7#79 | 2009 |
| ERP004677 | PRJEB5280 | ERZ369498 | ERS519170 | FPTC01000001-FPTC01000077 | 14722_7#8 | 2011 |
| ERP004677 | PRJEB5280 | ERZ369499 | ERS518810 | FPTD01000001-FPTD01000077 | 14722_7#80 | 2009 |
| ERP004677 | PRJEB5280 | ERZ369500 | ERS518811 | FPTE01000001-FPTE01000074 | 14722_7#81 | 2009 |
| ERP004677 | PRJEB5280 | ERZ369501 | ERS518812 | FPTH01000001-FPTH01000071 | 14722_7#82 | 2009 |
| ERP004677 | PRJEB5280 | ERZ369502 | ERS518813 | FQOT01000001-FQOT01000069 | 14722_7#83 | 2009 |
| ERP004677 | PRJEB5280 | ERZ369503 | ERS518814 | FQNX01000001-FQNX01000086 | 14722_7#84 | 2009 |
| ERP004677 | PRJEB5280 | ERZ369504 | ERS518815 | FPTF01000001-FPTF01000067 | 14722_7#85 | 2009 |
| ERP004677 | PRJEB5280 | ERZ369505 | ERS518816 | FPTG01000001-FPTG01000063 | 14722_7#86 | 2009 |
| ERP004677 | PRJEB5280 | ERZ369506 | ERS518817 | FPTI01000001-FPTI01000071 | 14722_7#87 | 2009 |
| ERP004677 | PRJEB5280 | ERZ369507 | ERS518818 | FPTJ01000001-FPTJ01000072 | 14722_7#88 | 2009 |
| ERP004677 | PRJEB5280 | ERZ369508 | ERS518820 | FPTL01000001-FPTL01000070 | 14722_7#89 | 2009 |
| ERP004677 | PRJEB5280 | ERZ369509 | ERS519176 | FPTK01000001-FPTK01000088 | 14722_7#9 | 2012 |
| ERP004677 | PRJEB5280 | ERZ369510 | ERS518821 | FPTM01000001-FPTM01000070 | 14722_7#90 | 2009 |
| ERP004677 | PRJEB5280 | ERZ369511 | ERS518824 | FPTN01000001-FPTN01000086 | 14722_7#91 | 2009 |
| ERP004677 | PRJEB5280 | ERZ369512 | ERS518825 | FPTP01000001-FPTP01000084 | 14722_7#92 | 2009 |
| ERP004677 | PRJEB5280 | ERZ369513 | ERS518826 | FPTQ01000001-FPTQ01000075 | 14722_7#93 | 2009 |
| ERP004677 | PRJEB5280 | ERZ369514 | ERS518827 | FPTO01000001-FPTO01000090 | 14722_7#94 | 2009 |
| ERP004677 | PRJEB5280 | ERZ369515 | ERS518834 | FPTR01000001-FPTR01000074 | 14722_7#95 | 2009 |
| ERP004677 | PRJEB5280 | ERZ369516 | ERS519074 | FPTS01000001-FPTS01000071 | 14722_8#1 | 2011 |
| ERP004677 | PRJEB5280 | ERZ369517 | ERS519089 | FPTT01000001-FPTT01000067 | 14722_8#10 | 2011 |
| ERP004677 | PRJEB5280 | ERZ369518 | ERS519090 | FPTV01000001-FPTV01000082 | 14722_8#11 | 2011 |
| ERP004677 | PRJEB5280 | ERZ369519 | ERS519092 | FPTU01000001-FPTU01000080 | 14722_8#12 | 2010 |
| ERP004677 | PRJEB5280 | ERZ369520 | ERS519093 | FPTW01000001-FPTW01000085 | 14722_8#13 | 2011 |
| ERP004677 | PRJEB5280 | ERZ369521 | ERS519094 | FPTX01000001-FPTX01000083 | 14722_8#14 | 2011 |
| ERP004677 | PRJEB5280 | ERZ369522 | ERS519095 | FPTY01000001-FPTY01000075 | 14722_8#15 | 2011 |
| ERP004677 | PRJEB5280 | ERZ369523 | ERS519096 | FPTZ01000001-FPTZ01000449 | 14722_8#16 | 2011 |
| ERP004677 | PRJEB5280 | ERZ369524 | ERS519097 | FPUA01000001-FPUA01000083 | 14722_8#17 | 2011 |
| ERP004677 | PRJEB5280 | ERZ369525 | ERS519098 | FQOV01000001-FQOV01000078 | 14722_8#18 | 2011 |
| ERP004677 | PRJEB5280 | ERZ369526 | ERS519099 | FQOX01000001-FQOX01000089 | 14722_8#19 | 2011 |
| ERP004677 | PRJEB5280 | ERZ369527 | ERS519075 | FPUB01000001-FPUB01000084 | 14722_8#2 | 2011 |
| ERP004677 | PRJEB5280 | ERZ369528 | ERS519100 | FPUC01000001-FPUC01000073 | 14722_8#20 | 2011 |
| ERP004677 | PRJEB5280 | ERZ369529 | ERS519101 | FPUD01000001-FPUD01000086 | 14722_8#21 | 2011 |
| ERP004677 | PRJEB5280 | ERZ369530 | ERS519102 | FPUE01000001-FPUE01000079 | 14722_8#22 | 2011 |
| ERP004677 | PRJEB5280 | ERZ369531 | ERS519103 | FPUF01000001-FPUF01000077 | 14722_8#23 | 2010 |
| ERP004677 | PRJEB5280 | ERZ369532 | ERS519104 | FPUG01000001-FPUG01000080 | 14722_8#24 | 2010 |
| ERP004677 | PRJEB5280 | ERZ369533 | ERS519105 | FPUH01000001-FPUH01000075 | 14722_8#25 | 2011 |
| ERP004677 | PRJEB5280 | ERZ369534 | ERS519106 | FPUI01000001-FPUI01000077 | 14722_8#26 | 2011 |
| ERP004677 | PRJEB5280 | ERZ369535 | ERS519107 | FPUJ01000001-FPUJ01000084 | 14722_8#27 | 2011 |
| ERP004677 | PRJEB5280 | ERZ369536 | ERS519108 | FPUK01000001-FPUK01000090 | 14722_8#28 | 2011 |
| ERP004677 | PRJEB5280 | ERZ369537 | ERS519109 | FQOP01000001-FQOP01000087 | 14722_8#29 | 2011 |
| ERP004677 | PRJEB5280 | ERZ369538 | ERS519077 | FQOQ01000001-FQOQ01000075 | 14722_8#3 | 2011 |
| ERP004677 | PRJEB5280 | ERZ369539 | ERS519110 | FPUL01000001-FPUL01000406 | 14722_8#30 | 2011 |
| ERP004677 | PRJEB5280 | ERZ369540 | ERS519111 | FPUN01000001-FPUN01000078 | 14722_8#31 | 2011 |
| ERP004677 | PRJEB5280 | ERZ369541 | ERS519112 | FPUM01000001-FPUM01000083 | 14722_8#32 | 2011 |
| ERP004677 | PRJEB5280 | ERZ369542 | ERS519113 | FPUP01000001-FPUP01000073 | 14722_8#33 | 2011 |
| ERP004677 | PRJEB5280 | ERZ369543 | ERS519114 | FPUO01000001-FPUO01000088 | 14722_8#34 | 2011 |
| ERP004677 | PRJEB5280 | ERZ369544 | ERS519115 | FPUQ01000001-FPUQ01000076 | 14722_8#35 | 2011 |
| ERP004677 | PRJEB5280 | ERZ369545 | ERS519116 | FPUS01000001-FPUS01000076 | 14722_8#36 | 2011 |
| ERP004677 | PRJEB5280 | ERZ369546 | ERS519118 | FPUR01000001-FPUR01000069 | 14722_8#37 | 2011 |
| ERP004677 | PRJEB5280 | ERZ369547 | ERS519119 | FQOC01000001-FQOC01000076 | 14722_8#38 | 2011 |
| ERP004677 | PRJEB5280 | ERZ369548 | ERS519121 | FPUT01000001-FPUT01000087 | 14722_8#39 | 2011 |
| ERP004677 | PRJEB5280 | ERZ369549 | ERS519078 | FPUU01000001-FPUU01000065 | 14722_8#4 | 2011 |
| ERP004677 | PRJEB5280 | ERZ369550 | ERS519122 | FPUV01000001-FPUV01000084 | 14722_8#40 | 2011 |
| ERP004677 | PRJEB5280 | ERZ369551 | ERS519124 | FPUX01000001-FPUX01000071 | 14722_8#41 | 2011 |
| ERP004677 | PRJEB5280 | ERZ369552 | ERS519125 | FPUW01000001-FPUW01000088 | 14722_8#42 | 2011 |
| ERP004677 | PRJEB5280 | ERZ369553 | ERS519126 | FPVB01000001-FPVB01000075 | 14722_8#43 | 2011 |
| ERP004677 | PRJEB5280 | ERZ369554 | ERS519127 | FPUY01000001-FPUY01000073 | 14722_8#44 | 2011 |
| ERP004677 | PRJEB5280 | ERZ369555 | ERS519128 | FPUZ01000001-FPUZ01000074 | 14722_8#45 | 2011 |
| ERP004677 | PRJEB5280 | ERZ369556 | ERS519129 | FPVA01000001-FPVA01000079 | 14722_8#46 | 2011 |
| ERP004677 | PRJEB5280 | ERZ369557 | ERS519130 | FPVC01000001-FPVC01000085 | 14722_8#47 | 2011 |
| ERP004677 | PRJEB5280 | ERZ369558 | ERS519131 | FPVD01000001-FPVD01000088 | 14722_8#48 | 2011 |
| ERP004677 | PRJEB5280 | ERZ369559 | ERS519132 | FPVF01000001-FPVF01000089 | 14722_8#49 | 2011 |
| ERP004677 | PRJEB5280 | ERZ369560 | ERS519080 | FPVE01000001-FPVE01000076 | 14722_8#5 | 2011 |
| ERP004677 | PRJEB5280 | ERZ369561 | ERS519136 | FPVG01000001-FPVG01000092 | 14722_8#50 | 2011 |
| ERP004677 | PRJEB5280 | ERZ369562 | ERS519137 | FQOK01000001-FQOK01000074 | 14722_8#51 | 2011 |
| ERP004677 | PRJEB5280 | ERZ369563 | ERS519138 | FPVH01000001-FPVH01000076 | 14722_8#52 | 2011 |
| ERP004677 | PRJEB5280 | ERZ369564 | ERS519139 | FPVI01000001-FPVI01000075 | 14722_8#53 | 2011 |
| ERP004677 | PRJEB5280 | ERZ369565 | ERS519140 | FPVK01000001-FPVK01000081 | 14722_8#54 | 2010 |
| ERP004677 | PRJEB5280 | ERZ369566 | ERS519142 | FPVJ01000001-FPVJ01000086 | 14722_8#55 | 2011 |
| ERP004677 | PRJEB5280 | ERZ369567 | ERS519143 | FPVL01000001-FPVL01000082 | 14722_8#56 | 2011 |
| ERP004677 | PRJEB5280 | ERZ369568 | ERS519144 | FPVN01000001-FPVN01000095 | 14722_8#57 | 2011 |
| ERP004677 | PRJEB5280 | ERZ369569 | ERS519145 | FPVM01000001-FPVM01000072 | 14722_8#58 | 2012 |
| ERP004677 | PRJEB5280 | ERZ369570 | ERS519147 | FPVO01000001-FPVO01000087 | 14722_8#59 | 2012 |
| ERP004677 | PRJEB5280 | ERZ369571 | ERS519081 | FPVP01000001-FPVP01000072 | 14722_8#6 | 2011 |
| ERP004677 | PRJEB5280 | ERZ369572 | ERS519148 | FPVQ01000001-FPVQ01000071 | 14722_8#60 | 2011 |
| ERP004677 | PRJEB5280 | ERZ369573 | ERS519150 | FPVR01000001-FPVR01000076 | 14722_8#61 | 2012 |
| ERP004677 | PRJEB5280 | ERZ369574 | ERS519151 | FPVS01000001-FPVS01000081 | 14722_8#62 | 2008 |
| ERP004677 | PRJEB5280 | ERZ369575 | ERS519152 | FPVT01000001-FPVT01000078 | 14722_8#63 | 2011 |
| ERP004677 | PRJEB5280 | ERZ369576 | ERS519153 | FPVV01000001-FPVV01000091 | 14722_8#64 | 2007 |
| ERP004677 | PRJEB5280 | ERZ369577 | ERS519154 | FPVU01000001-FPVU01000078 | 14722_8#65 | 2012 |
| ERP004677 | PRJEB5280 | ERZ369578 | ERS519155 | FPVW01000001-FPVW01000086 | 14722_8#66 | 2011 |
| ERP004677 | PRJEB5280 | ERZ369579 | ERS519156 | FPVX01000001-FPVX01000086 | 14722_8#67 | 2011 |
| ERP004677 | PRJEB5280 | ERZ369580 | ERS519157 | FPVY01000001-FPVY01000088 | 14722_8#68 | 2011 |
| ERP004677 | PRJEB5280 | ERZ369581 | ERS519159 | FQNY01000001-FQNY01000080 | 14722_8#69 | 2012 |
| ERP004677 | PRJEB5280 | ERZ369582 | ERS519082 | FPVZ01000001-FPVZ01000092 | 14722_8#7 | 2011 |
| ERP004677 | PRJEB5280 | ERZ369583 | ERS519160 | FPWB01000001-FPWB01000082 | 14722_8#70 | 2012 |
| ERP004677 | PRJEB5280 | ERZ369584 | ERS519161 | FPWA01000001-FPWA01000089 | 14722_8#71 | 2011 |
| ERP004677 | PRJEB5280 | ERZ369585 | ERS519162 | FPWC01000001-FPWC01000070 | 14722_8#72 | 2011 |
| ERP004677 | PRJEB5280 | ERZ369586 | ERS519163 | FPWD01000001-FPWD01000076 | 14722_8#73 | 2011 |
| ERP004677 | PRJEB5280 | ERZ369587 | ERS519164 | FPWE01000001-FPWE01000071 | 14722_8#74 | 2011 |
| ERP004677 | PRJEB5280 | ERZ369588 | ERS519165 | FPWF01000001-FPWF01000075 | 14722_8#75 | 2012 |
| ERP004677 | PRJEB5280 | ERZ369589 | ERS519166 | FPWG01000001-FPWG01000067 | 14722_8#76 | 2012 |
| ERP004677 | PRJEB5280 | ERZ369590 | ERS519167 | FPWH01000001-FPWH01000088 | 14722_8#77 | 2012 |
| ERP004677 | PRJEB5280 | ERZ369591 | ERS519169 | FPWJ01000001-FPWJ01000088 | 14722_8#78 | 2012 |
| ERP004677 | PRJEB5280 | ERZ369592 | ERS519171 | FPWI01000001-FPWI01000073 | 14722_8#79 | 2012 |
| ERP004677 | PRJEB5280 | ERZ369593 | ERS519084 | FPWK01000001-FPWK01000077 | 14722_8#8 | 2011 |
| ERP004677 | PRJEB5280 | ERZ369594 | ERS519172 | FPWL01000001-FPWL01000073 | 14722_8#80 | 2012 |
| ERP004677 | PRJEB5280 | ERZ369595 | ERS519173 | FPWN01000001-FPWN01000084 | 14722_8#81 | 2012 |
| ERP004677 | PRJEB5280 | ERZ369596 | ERS519174 | FQOH01000001-FQOH01000086 | 14722_8#82 | 2012 |
| ERP004677 | PRJEB5280 | ERZ369597 | ERS519175 | FPWM01000001-FPWM01000083 | 14722_8#83 | 2012 |
| ERP004677 | PRJEB5280 | ERZ369598 | ERS519180 | FPWP01000001-FPWP01000082 | 14722_8#84 | 2012 |
| ERP004677 | PRJEB5280 | ERZ369599 | ERS519181 | FPWO01000001-FPWO01000081 | 14722_8#85 | 2012 |
| ERP004677 | PRJEB5280 | ERZ369600 | ERS519183 | FPWQ01000001-FPWQ01000074 | 14722_8#86 | 2012 |
| ERP004677 | PRJEB5280 | ERZ369601 | ERS519186 | FPWR01000001-FPWR01000084 | 14722_8#87 | 2012 |
| ERP004677 | PRJEB5280 | ERZ369602 | ERS519190 | FPWU01000001-FPWU01000087 | 14722_8#88 | 2012 |
| ERP004677 | PRJEB5280 | ERZ369603 | ERS519191 | FPWS01000001-FPWS01000078 | 14722_8#89 | 2012 |
| ERP004677 | PRJEB5280 | ERZ369604 | ERS519088 | FPWT01000001-FPWT01000078 | 14722_8#9 | 2006 |
| ERP004677 | PRJEB5280 | ERZ369605 | ERS519192 | FQOA01000001-FQOA01000090 | 14722_8#90 | 2012 |
| ERP004677 | PRJEB5280 | ERZ369606 | ERS519200 | FPWW01000001-FPWW01000084 | 14722_8#91 | 2012 |
| ERP004677 | PRJEB5280 | ERZ369607 | ERS519201 | FQOY01000001-FQOY01000089 | 14722_8#92 | 2011 |
| ERP004677 | PRJEB5280 | ERZ369608 | ERS519202 | FPWV01000001-FPWV01000069 | 14722_8#93 | 2011 |
| ERP004677 | PRJEB5280 | ERZ369609 | ERS519203 | FPXC01000001-FPXC01000092 | 14722_8#94 | 2011 |
| ERP004677 | PRJEB5280 | ERZ369610 | ERS519205 | FQOE01000001-FQOE01000070 | 14722_8#95 | 2012 |
| ERP004677 | PRJEB5280 | ERZ369611 | ERS518835 | FPWX01000001-FPWX01000079 | 14892_2#1 | 2009 |
| ERP004677 | PRJEB5280 | ERZ369612 | ERS518853 | FPWY01000001-FPWY01000063 | 14892_2#10 | 2009 |
| ERP004677 | PRJEB5280 | ERZ369613 | ERS518854 | FPXA01000001-FPXA01000084 | 14892_2#11 | 2009 |
| ERP004677 | PRJEB5280 | ERZ369614 | ERS518861 | FPXB01000001-FPXB01000084 | 14892_2#12 | 2009 |
| ERP004677 | PRJEB5280 | ERZ369615 | ERS518866 | FPWZ01000001-FPWZ01000057 | 14892_2#15 | 2009 |
| ERP004677 | PRJEB5280 | ERZ369616 | ERS518868 | FPXF01000001-FPXF01000070 | 14892_2#16 | 2009 |
| ERP004677 | PRJEB5280 | ERZ369617 | ERS518869 | FPXE01000001-FPXE01000069 | 14892_2#17 | 2009 |
| ERP004677 | PRJEB5280 | ERZ369618 | ERS518870 | FPXG01000001-FPXG01000070 | 14892_2#18 | 2009 |
| ERP004677 | PRJEB5280 | ERZ369619 | ERS518841 | FPXD01000001-FPXD01000072 | 14892_2#2 | 2009 |
| ERP004677 | PRJEB5280 | ERZ369620 | ERS518872 | FPXJ01000001-FPXJ01000075 | 14892_2#20 | 2009 |
| ERP004677 | PRJEB5280 | ERZ369621 | ERS518875 | FPXH01000001-FPXH01000082 | 14892_2#21 | 2009 |
| ERP004677 | PRJEB5280 | ERZ369622 | ERS518876 | FPXI01000001-FPXI01000057 | 14892_2#22 | 2009 |
| ERP004677 | PRJEB5280 | ERZ369623 | ERS518879 | FQNR01000001-FQNR01000081 | 14892_2#23 | 2009 |
| ERP004677 | PRJEB5280 | ERZ369624 | ERS518883 | FPXL01000001-FPXL01000086 | 14892_2#25 | 2009 |
| ERP004677 | PRJEB5280 | ERZ369625 | ERS518886 | FPXK01000001-FPXK01000066 | 14892_2#26 | 2009 |
| ERP004677 | PRJEB5280 | ERZ369626 | ERS518887 | FPXM01000001-FPXM01000069 | 14892_2#27 | 2009 |
| ERP004677 | PRJEB5280 | ERZ369627 | ERS518889 | FPXO01000001-FPXO01000071 | 14892_2#28 | 2009 |
| ERP004677 | PRJEB5280 | ERZ369628 | ERS518890 | FQOG01000001-FQOG01000060 | 14892_2#29 | 2009 |
| ERP004677 | PRJEB5280 | ERZ369629 | ERS518891 | FPXN01000001-FPXN01000073 | 14892_2#30 | 2009 |
| ERP004677 | PRJEB5280 | ERZ369630 | ERS518892 | FPXP01000001-FPXP01000072 | 14892_2#31 | 2009 |
| ERP004677 | PRJEB5280 | ERZ369631 | ERS518893 | FPXQ01000001-FPXQ01000068 | 14892_2#32 | 2009 |
| ERP004677 | PRJEB5280 | ERZ369632 | ERS518898 | FPXR01000001-FPXR01000061 | 14892_2#33 | 2009 |
| ERP004677 | PRJEB5280 | ERZ369633 | ERS518900 | FPXS01000001-FPXS01000055 | 14892_2#34 | 2009 |
| ERP004677 | PRJEB5280 | ERZ369634 | ERS518901 | FPXT01000001-FPXT01000074 | 14892_2#35 | 2009 |
| ERP004677 | PRJEB5280 | ERZ369635 | ERS518908 | FPXV01000001-FPXV01000065 | 14892_2#36 | 2009 |
| ERP004677 | PRJEB5280 | ERZ369636 | ERS518909 | FPXU01000001-FPXU01000067 | 14892_2#37 | 2009 |
| ERP004677 | PRJEB5280 | ERZ369637 | ERS518916 | FQOO01000001-FQOO01000078 | 14892_2#39 | 2009 |
| ERP004677 | PRJEB5280 | ERZ369638 | ERS518844 | FPXW01000001-FPXW01000073 | 14892_2#4 | 2009 |
| ERP004677 | PRJEB5280 | ERZ369639 | ERS518925 | FPXX01000001-FPXX01000079 | 14892_2#40 | 2009 |
| ERP004677 | PRJEB5280 | ERZ369640 | ERS518930 | FQNV01000001-FQNV01000057 | 14892_2#41 | 2009 |
| ERP004677 | PRJEB5280 | ERZ369641 | ERS518937 | FPXY01000001-FPXY01000068 | 14892_2#42 | 2009 |
| ERP004677 | PRJEB5280 | ERZ369642 | ERS518940 | FPXZ01000001-FPXZ01000069 | 14892_2#43 | 2009 |
| ERP004677 | PRJEB5280 | ERZ369643 | ERS518947 | FPYA01000001-FPYA01000067 | 14892_2#44 | 2009 |
| ERP004677 | PRJEB5280 | ERZ369644 | ERS518953 | FPYB01000001-FPYB01000079 | 14892_2#45 | 2010 |
| ERP004677 | PRJEB5280 | ERZ369645 | ERS518957 | FQNM01000001-FQNM01000075 | 14892_2#46 | 2010 |
| ERP004677 | PRJEB5280 | ERZ369646 | ERS518967 | FPYC01000001-FPYC01000069 | 14892_2#48 | 2010 |
| ERP004677 | PRJEB5280 | ERZ369647 | ERS518972 | FPYD01000001-FPYD01000064 | 14892_2#49 | 2010 |
| ERP004677 | PRJEB5280 | ERZ369648 | ERS518847 | FPYE01000001-FPYE01000074 | 14892_2#5 | 2009 |
| ERP004677 | PRJEB5280 | ERZ369649 | ERS518978 | FPYF01000001-FPYF01000070 | 14892_2#50 | 2009 |
| ERP004677 | PRJEB5280 | ERZ369650 | ERS518980 | FPYH01000001-FPYH01000067 | 14892_2#51 | 2009 |
| ERP004677 | PRJEB5280 | ERZ369651 | ERS518981 | FPYI01000001-FPYI01000069 | 14892_2#52 | 2010 |
| ERP004677 | PRJEB5280 | ERZ369652 | ERS518987 | FPYG01000001-FPYG01000071 | 14892_2#53 | 2009 |
| ERP004677 | PRJEB5280 | ERZ369653 | ERS518993 | FPYJ01000001-FPYJ01000059 | 14892_2#54 | 2010 |
| ERP004677 | PRJEB5280 | ERZ369654 | ERS519006 | FPYL01000001-FPYL01000057 | 14892_2#57 | 2009 |
| ERP004677 | PRJEB5280 | ERZ369655 | ERS519007 | FPYK01000001-FPYK01000076 | 14892_2#58 | 2009 |
| ERP004677 | PRJEB5280 | ERZ369656 | ERS519009 | FPYM01000001-FPYM01000085 | 14892_2#59 | 2009 |
| ERP004677 | PRJEB5280 | ERZ369657 | ERS519010 | FPYN01000001-FPYN01000073 | 14892_2#60 | 2009 |
| ERP004677 | PRJEB5280 | ERZ369658 | ERS519012 | FQNG01000001-FQNG01000061 | 14892_2#61 | 2009 |
| ERP004677 | PRJEB5280 | ERZ369659 | ERS519024 | FPYO01000001-FPYO01000085 | 14892_2#63 | 2011 |
| ERP004677 | PRJEB5280 | ERZ369660 | ERS519025 | FPYP01000001-FPYP01000065 | 14892_2#64 | 2011 |
| ERP004677 | PRJEB5280 | ERZ369661 | ERS519027 | FPYQ01000001-FPYQ01000063 | 14892_2#65 | 2010 |
| ERP004677 | PRJEB5280 | ERZ369662 | ERS519029 | FPYR01000001-FPYR01000076 | 14892_2#66 | 2011 |
| ERP004677 | PRJEB5280 | ERZ369663 | ERS519030 | FPYS01000001-FPYS01000068 | 14892_2#67 | 2011 |
| ERP004677 | PRJEB5280 | ERZ369664 | ERS519031 | FPYT01000001-FPYT01000060 | 14892_2#68 | 2009 |
| ERP004677 | PRJEB5280 | ERZ369665 | ERS519032 | FPYU01000001-FPYU01000073 | 14892_2#69 | 2010 |
| ERP004677 | PRJEB5280 | ERZ369666 | ERS518849 | FPYV01000001-FPYV01000073 | 14892_2#7 | 2009 |
| ERP004677 | PRJEB5280 | ERZ369667 | ERS519033 | FPYX01000001-FPYX01000078 | 14892_2#70 | 2010 |
| ERP004677 | PRJEB5280 | ERZ369668 | ERS519034 | FPYZ01000001-FPYZ01000073 | 14892_2#71 | 2010 |
| ERP004677 | PRJEB5280 | ERZ369669 | ERS519035 | FPZA01000001-FPZA01000073 | 14892_2#72 | 2009 |
| ERP004677 | PRJEB5280 | ERZ369670 | ERS519036 | FPYW01000001-FPYW01000072 | 14892_2#73 | 2010 |
| ERP004677 | PRJEB5280 | ERZ369671 | ERS519037 | FPYY01000001-FPYY01000071 | 14892_2#74 | 2011 |
| ERP004677 | PRJEB5280 | ERZ369672 | ERS519039 | FPZC01000001-FPZC01000074 | 14892_2#75 | 2009 |
| ERP004677 | PRJEB5280 | ERZ369673 | ERS519040 | FQOW01000001-FQOW01000068 | 14892_2#76 | 2011 |
| ERP004677 | PRJEB5280 | ERZ369674 | ERS519047 | FPZB01000001-FPZB01000079 | 14892_2#77 | 2011 |
| ERP004677 | PRJEB5280 | ERZ369675 | ERS519050 | FPZD01000001-FPZD01000088 | 14892_2#78 | 2011 |
| ERP004677 | PRJEB5280 | ERZ369676 | ERS519051 | FPZG01000001-FPZG01000095 | 14892_2#79 | 2011 |
| ERP004677 | PRJEB5280 | ERZ369677 | ERS518850 | FPZF01000001-FPZF01000074 | 14892_2#8 | 2009 |
| ERP004677 | PRJEB5280 | ERZ369678 | ERS519052 | FQOB01000001-FQOB01000070 | 14892_2#80 | 2011 |
| ERP004677 | PRJEB5280 | ERZ369679 | ERS519053 | FPZH01000001-FPZH01000080 | 14892_2#81 | 2011 |
| ERP004677 | PRJEB5280 | ERZ369680 | ERS519055 | FPZK01000001-FPZK01000501 | 14892_2#82 | 2011 |
| ERP004677 | PRJEB5280 | ERZ369681 | ERS519056 | FPZE01000001-FPZE01000089 | 14892_2#83 | 2011 |
| ERP004677 | PRJEB5280 | ERZ369682 | ERS519057 | FQOJ01000001-FQOJ01000067 | 14892_2#84 | 2011 |
| ERP004677 | PRJEB5280 | ERZ369683 | ERS519058 | FPZJ01000001-FPZJ01000067 | 14892_2#85 | 2011 |
| ERP004677 | PRJEB5280 | ERZ369684 | ERS519059 | FPZI01000001-FPZI01000080 | 14892_2#86 | 2011 |
| ERP004677 | PRJEB5280 | ERZ369685 | ERS519062 | FPZN01000001-FPZN01000063 | 14892_2#88 | 2011 |
| ERP004677 | PRJEB5280 | ERZ369686 | ERS519063 | FPZO01000001-FPZO01000074 | 14892_2#89 | 2011 |
| ERP004677 | PRJEB5280 | ERZ369687 | ERS518852 | FPZL01000001-FPZL01000087 | 14892_2#9 | 2009 |
| ERP004677 | PRJEB5280 | ERZ369688 | ERS519064 | FPZP01000001-FPZP01000070 | 14892_2#90 | 2011 |
| ERP004677 | PRJEB5280 | ERZ369689 | ERS519065 | FPZM01000001-FPZM01000083 | 14892_2#91 | 2011 |
| ERP004677 | PRJEB5280 | ERZ369690 | ERS519066 | FPZQ01000001-FPZQ01000084 | 14892_2#92 | 2011 |
| ERP004677 | PRJEB5280 | ERZ369691 | ERS519069 | FPZR01000001-FPZR01000060 | 14892_2#93 | 2011 |
| ERP004677 | PRJEB5280 | ERZ369692 | ERS519070 | FPZS01000001-FPZS01000059 | 14892_2#94 | 2011 |
| ERP004677 | PRJEB5280 | ERZ369693 | ERS519072 | FPZT01000001-FPZT01000074 | 14892_2#95 | 2011 |
| ERP004677 | PRJEB5280 | ERZ369694 | ERS518936 | FPZU01000001-FPZU01000068 | 14893_2#1 | 2009 |
| ERP004677 | PRJEB5280 | ERZ369695 | ERS518948 | FQNO01000001-FQNO01000072 | 14893_2#10 | 2009 |
| ERP004677 | PRJEB5280 | ERZ369696 | ERS518949 | FPZW01000001-FPZW01000064 | 14893_2#11 | 2010 |
| ERP004677 | PRJEB5280 | ERZ369697 | ERS518950 | FQOI01000001-FQOI01000071 | 14893_2#12 | 2009 |
| ERP004677 | PRJEB5280 | ERZ369698 | ERS518951 | FPZV01000001-FPZV01000067 | 14893_2#13 | 2010 |
| ERP004677 | PRJEB5280 | ERZ369699 | ERS518952 | FPZX01000001-FPZX01000072 | 14893_2#14 | 2009 |
| ERP004677 | PRJEB5280 | ERZ369700 | ERS518954 | FQNW01000001-FQNW01000082 | 14893_2#15 | 2010 |
| ERP004677 | PRJEB5280 | ERZ369701 | ERS518955 | FPZY01000001-FPZY01000067 | 14893_2#16 | 2010 |
| ERP004677 | PRJEB5280 | ERZ369702 | ERS518956 | FPZZ01000001-FPZZ01000068 | 14893_2#17 | 2010 |
| ERP004677 | PRJEB5280 | ERZ369703 | ERS518958 | FQAA01000001-FQAA01000070 | 14893_2#18 | 2009 |
| ERP004677 | PRJEB5280 | ERZ369704 | ERS518959 | FQAC01000001-FQAC01000076 | 14893_2#19 | 2010 |
| ERP004677 | PRJEB5280 | ERZ369705 | ERS518938 | FQAB01000001-FQAB01000057 | 14893_2#2 | 2010 |
| ERP004677 | PRJEB5280 | ERZ369706 | ERS518960 | FQAE01000001-FQAE01000065 | 14893_2#20 | 2009 |
| ERP004677 | PRJEB5280 | ERZ369707 | ERS518961 | FQOD01000001-FQOD01000064 | 14893_2#21 | 2009 |
| ERP004677 | PRJEB5280 | ERZ369708 | ERS518962 | FQAD01000001-FQAD01000071 | 14893_2#22 | 2010 |
| ERP004677 | PRJEB5280 | ERZ369709 | ERS518963 | FQNJ01000001-FQNJ01000075 | 14893_2#23 | 2009 |
| ERP004677 | PRJEB5280 | ERZ369710 | ERS518965 | FQOR01000001-FQOR01000082 | 14893_2#24 | 2009 |
| ERP004677 | PRJEB5280 | ERZ369711 | ERS518966 | FQAF01000001-FQAF01000079 | 14893_2#25 | 2010 |
| ERP004677 | PRJEB5280 | ERZ369712 | ERS518968 | FQAH01000001-FQAH01000065 | 14893_2#26 | 2010 |
| ERP004677 | PRJEB5280 | ERZ369713 | ERS518969 | FQAG01000001-FQAG01000077 | 14893_2#27 | 2010 |
| ERP004677 | PRJEB5280 | ERZ369714 | ERS518970 | FQSU01000001-FQSU01000084 | 14893_2#28 | NA |
| ERP004677 | PRJEB5280 | ERZ369715 | ERS518971 | FQOM01000001-FQOM01000069 | 14893_2#29 | 2009 |
| ERP004677 | PRJEB5280 | ERZ369716 | ERS518939 | FQNT01000001-FQNT01000064 | 14893_2#3 | 2010 |
| ERP004677 | PRJEB5280 | ERZ369717 | ERS518973 | FQAI01000001-FQAI01000066 | 14893_2#30 | 2010 |
| ERP004677 | PRJEB5280 | ERZ369718 | ERS518974 | FQAK01000001-FQAK01000277 | 14893_2#31 | 2009 |
| ERP004677 | PRJEB5280 | ERZ369719 | ERS518975 | FQAJ01000001-FQAJ01000080 | 14893_2#32 | 2010 |
| ERP004677 | PRJEB5280 | ERZ369720 | ERS518976 | FQAL01000001-FQAL01000083 | 14893_2#33 | 2009 |
| ERP004677 | PRJEB5280 | ERZ369721 | ERS518977 | FQAM01000001-FQAM01000075 | 14893_2#34 | 2009 |
| ERP004677 | PRJEB5280 | ERZ369722 | ERS518979 | FQAN01000001-FQAN01000134 | 14893_2#35 | 2010 |
| ERP004677 | PRJEB5280 | ERZ369723 | ERS518982 | FQAO01000001-FQAO01000066 | 14893_2#36 | 2009 |
| ERP004677 | PRJEB5280 | ERZ369724 | ERS518983 | FQAP01000001-FQAP01000075 | 14893_2#37 | 2010 |
| ERP004677 | PRJEB5280 | ERZ369725 | ERS518984 | FQAQ01000001-FQAQ01000088 | 14893_2#38 | 2010 |
| ERP004677 | PRJEB5280 | ERZ369726 | ERS518985 | FQAR01000001-FQAR01000080 | 14893_2#39 | 2010 |
| ERP004677 | PRJEB5280 | ERZ369727 | ERS518941 | FQAS01000001-FQAS01000065 | 14893_2#4 | 2009 |
| ERP004677 | PRJEB5280 | ERZ369728 | ERS518986 | FQAT01000001-FQAT01000067 | 14893_2#40 | 2009 |
| ERP004677 | PRJEB5280 | ERZ369729 | ERS518988 | FQAV01000001-FQAV01000059 | 14893_2#41 | 2009 |
| ERP004677 | PRJEB5280 | ERZ369730 | ERS518989 | FQOS01000001-FQOS01000068 | 14893_2#42 | 2009 |
| ERP004677 | PRJEB5280 | ERZ369731 | ERS518990 | FQAU01000001-FQAU01000063 | 14893_2#43 | 2009 |
| ERP004677 | PRJEB5280 | ERZ369732 | ERS518991 | FQAW01000001-FQAW01000066 | 14893_2#44 | 2009 |
| ERP004677 | PRJEB5280 | ERZ369733 | ERS518992 | FQAX01000001-FQAX01000070 | 14893_2#45 | 2009 |
| ERP004677 | PRJEB5280 | ERZ369734 | ERS518994 | FQAY01000001-FQAY01000063 | 14893_2#46 | 2009 |
| ERP004677 | PRJEB5280 | ERZ369735 | ERS518995 | FQBA01000001-FQBA01000065 | 14893_2#47 | 2009 |
| ERP004677 | PRJEB5280 | ERZ369736 | ERS518996 | FQAZ01000001-FQAZ01000061 | 14893_2#48 | 2009 |
| ERP004677 | PRJEB5280 | ERZ369737 | ERS518997 | FQBC01000001-FQBC01000073 | 14893_2#49 | 2009 |
| ERP004677 | PRJEB5280 | ERZ369738 | ERS518942 | FQBB01000001-FQBB01000087 | 14893_2#5 | 2009 |
| ERP004677 | PRJEB5280 | ERZ369739 | ERS518998 | FQBE01000001-FQBE01000065 | 14893_2#50 | 2009 |
| ERP004677 | PRJEB5280 | ERZ369740 | ERS518999 | FQBD01000001-FQBD01000064 | 14893_2#51 | 2009 |
| ERP004677 | PRJEB5280 | ERZ369741 | ERS519001 | FQBF01000001-FQBF01000066 | 14893_2#52 | 2009 |
| ERP004677 | PRJEB5280 | ERZ369742 | ERS519002 | FQBG01000001-FQBG01000071 | 14893_2#53 | 2009 |
| ERP004677 | PRJEB5280 | ERZ369743 | ERS519003 | FQBI01000001-FQBI01000095 | 14893_2#54 | 2009 |
| ERP004677 | PRJEB5280 | ERZ369744 | ERS519004 | FQBK01000001-FQBK01000096 | 14893_2#55 | 2009 |
| ERP004677 | PRJEB5280 | ERZ369745 | ERS519008 | FQBH01000001-FQBH01000077 | 14893_2#56 | 2009 |
| ERP004677 | PRJEB5280 | ERZ369746 | ERS519011 | FQBJ01000001-FQBJ01000070 | 14893_2#57 | 2009 |
| ERP004677 | PRJEB5280 | ERZ369747 | ERS519013 | FQBM01000001-FQBM01000056 | 14893_2#58 | 2009 |
| ERP004677 | PRJEB5280 | ERZ369748 | ERS519014 | FQBL01000001-FQBL01000070 | 14893_2#59 | 2009 |
| ERP004677 | PRJEB5280 | ERZ369749 | ERS518943 | FQBN01000001-FQBN01000062 | 14893_2#6 | 2009 |
| ERP004677 | PRJEB5280 | ERZ369750 | ERS519015 | FQBO01000001-FQBO01000071 | 14893_2#60 | 2009 |
| ERP004677 | PRJEB5280 | ERZ369751 | ERS519016 | FQBQ01000001-FQBQ01000069 | 14893_2#61 | 2009 |
| ERP004677 | PRJEB5280 | ERZ369752 | ERS519017 | FQBP01000001-FQBP01000068 | 14893_2#62 | 2009 |
| ERP004677 | PRJEB5280 | ERZ369753 | ERS519018 | FQBR01000001-FQBR01000064 | 14893_2#63 | 2009 |
| ERP004677 | PRJEB5280 | ERZ369754 | ERS519019 | FQOF01000001-FQOF01000070 | 14893_2#64 | 2009 |
| ERP004677 | PRJEB5280 | ERZ369755 | ERS519020 | FQBS01000001-FQBS01000078 | 14893_2#65 | 2009 |
| ERP004677 | PRJEB5280 | ERZ369756 | ERS519022 | FQBT01000001-FQBT01000068 | 14893_2#66 | 2009 |
| ERP004677 | PRJEB5280 | ERZ369757 | ERS519023 | FQBU01000001-FQBU01000062 | 14893_2#67 | 2010 |
| ERP004677 | PRJEB5280 | ERZ369758 | ERS519026 | FQBV01000001-FQBV01000076 | 14893_2#68 | 2011 |
| ERP004677 | PRJEB5280 | ERZ369759 | ERS519028 | FQBW01000001-FQBW01000078 | 14893_2#69 | 2011 |
| ERP004677 | PRJEB5280 | ERZ369760 | ERS518944 | FQBX01000001-FQBX01000061 | 14893_2#7 | 2010 |
| ERP004677 | PRJEB5280 | ERZ369761 | ERS519038 | FQBY01000001-FQBY01000079 | 14893_2#70 | 2011 |
| ERP004677 | PRJEB5280 | ERZ369762 | ERS519041 | FQNZ01000001-FQNZ01000078 | 14893_2#71 | 2011 |
| ERP004677 | PRJEB5280 | ERZ369763 | ERS519042 | FQBZ01000001-FQBZ01000059 | 14893_2#72 | 2011 |
| ERP004677 | PRJEB5280 | ERZ369764 | ERS519043 | FQCB01000001-FQCB01000065 | 14893_2#73 | 2011 |
| ERP004677 | PRJEB5280 | ERZ369765 | ERS519044 | FQCA01000001-FQCA01000064 | 14893_2#74 | 2011 |
| ERP004677 | PRJEB5280 | ERZ369766 | ERS519045 | FQCC01000001-FQCC01000072 | 14893_2#75 | 2011 |
| ERP004677 | PRJEB5280 | ERZ369767 | ERS519046 | FQCD01000001-FQCD01000056 | 14893_2#76 | 2011 |
| ERP004677 | PRJEB5280 | ERZ369768 | ERS519048 | FQCE01000001-FQCE01000060 | 14893_2#77 | 2011 |
| ERP004677 | PRJEB5280 | ERZ369769 | ERS519049 | FQOU01000001-FQOU01000092 | 14893_2#78 | 2011 |
| ERP004677 | PRJEB5280 | ERZ369770 | ERS519054 | FQCF01000001-FQCF01000071 | 14893_2#79 | 2011 |
| ERP004677 | PRJEB5280 | ERZ369771 | ERS518945 | FQCG01000001-FQCG01000086 | 14893_2#8 | 2009 |
| ERP004677 | PRJEB5280 | ERZ369772 | ERS519061 | FQCH01000001-FQCH01000075 | 14893_2#80 | 2011 |
| ERP004677 | PRJEB5280 | ERZ369773 | ERS519067 | FQCI01000001-FQCI01000094 | 14893_2#81 | 2011 |
| ERP004677 | PRJEB5280 | ERZ369774 | ERS519068 | FQCJ01000001-FQCJ01000077 | 14893_2#82 | 2007 |
| ERP004677 | PRJEB5280 | ERZ369775 | ERS519071 | FQCK01000001-FQCK01000084 | 14893_2#83 | 2011 |
| ERP004677 | PRJEB5280 | ERZ369776 | ERS519073 | FQCL01000001-FQCL01000071 | 14893_2#84 | 2011 |
| ERP004677 | PRJEB5280 | ERZ369777 | ERS519076 | FQCM01000001-FQCM01000070 | 14893_2#85 | 2011 |
| ERP004677 | PRJEB5280 | ERZ369778 | ERS519079 | FQCN01000001-FQCN01000079 | 14893_2#86 | 2011 |
| ERP004677 | PRJEB5280 | ERZ369779 | ERS519083 | FQCO01000001-FQCO01000066 | 14893_2#87 | 2011 |
| ERP004677 | PRJEB5280 | ERZ369780 | ERS519085 | FQCP01000001-FQCP01000076 | 14893_2#88 | 2011 |
| ERP004677 | PRJEB5280 | ERZ369781 | ERS519086 | FQCQ01000001-FQCQ01000089 | 14893_2#89 | 2011 |
| ERP004677 | PRJEB5280 | ERZ369782 | ERS518946 | FQCR01000001-FQCR01000072 | 14893_2#9 | 2009 |
| ERP004677 | PRJEB5280 | ERZ369783 | ERS519087 | FQCS01000001-FQCS01000084 | 14893_2#90 | 2011 |
| ERP004677 | PRJEB5280 | ERZ369784 | ERS519091 | FQCU01000001-FQCU01000073 | 14893_2#91 | 2007 |
| ERP004677 | PRJEB5280 | ERZ369785 | ERS519117 | FQOZ01000001-FQOZ01000076 | 14893_2#92 | 2011 |
| ERP004677 | PRJEB5280 | ERZ369786 | ERS519120 | FQPA01000001-FQPA01000070 | 14893_2#93 | 2011 |
| ERP004677 | PRJEB5280 | ERZ369787 | ERS519123 | FQCT01000001-FQCT01000068 | 14893_2#94 | 2011 |
| ERP004677 | PRJEB5280 | ERZ369788 | ERS519133 | FQCV01000001-FQCV01000071 | 14893_2#95 | 2011 |
| ERP004677 | PRJEB5280 | ERZ369789 | ERS519207 | FQPB01000001-FQPB01000069 | 14893_3#65 | 2012 |
| ERP004677 | PRJEB5280 | ERZ369790 | ERS519208 | FQPF01000001-FQPF01000085 | 14893_3#66 | 2012 |
| ERP004677 | PRJEB5280 | ERZ369791 | ERS518865 | FQCW01000001-FQCW01000064 | 15277_3#50 | 2009 |
| ERP004677 | PRJEB5280 | ERZ369792 | ERS518871 | FQSW01000001-FQSW01000069 | 15277_3#51 | NA |
| ERP004677 | PRJEB5280 | ERZ369793 | ERS518882 | FQCX01000001-FQCX01000066 | 15277_3#52 | 2009 |
| ERP004677 | PRJEB5280 | ERZ369794 | ERS518911 | FQCY01000001-FQCY01000064 | 15277_3#53 | 2009 |
| ERP004677 | PRJEB5280 | ERZ369795 | ERS519000 | FQSV01000001-FQSV01000072 | 15277_3#55 | NA |
| ERP004677 | PRJEB5280 | ERZ369796 | ERS519021 | FQCZ01000001-FQCZ01000063 | 15277_3#57 | 2009 |
